# Supplementary figures and images for: Forkhead box D subfamily genes in colorectal cancer: potential biomarkers and therapeutic targets
Source: PeerJ. 2024 Oct 29;12:e18406. doi: 10.7717/peerj.18406 (PMC11529599; doi:10.7717/peerj.18406)

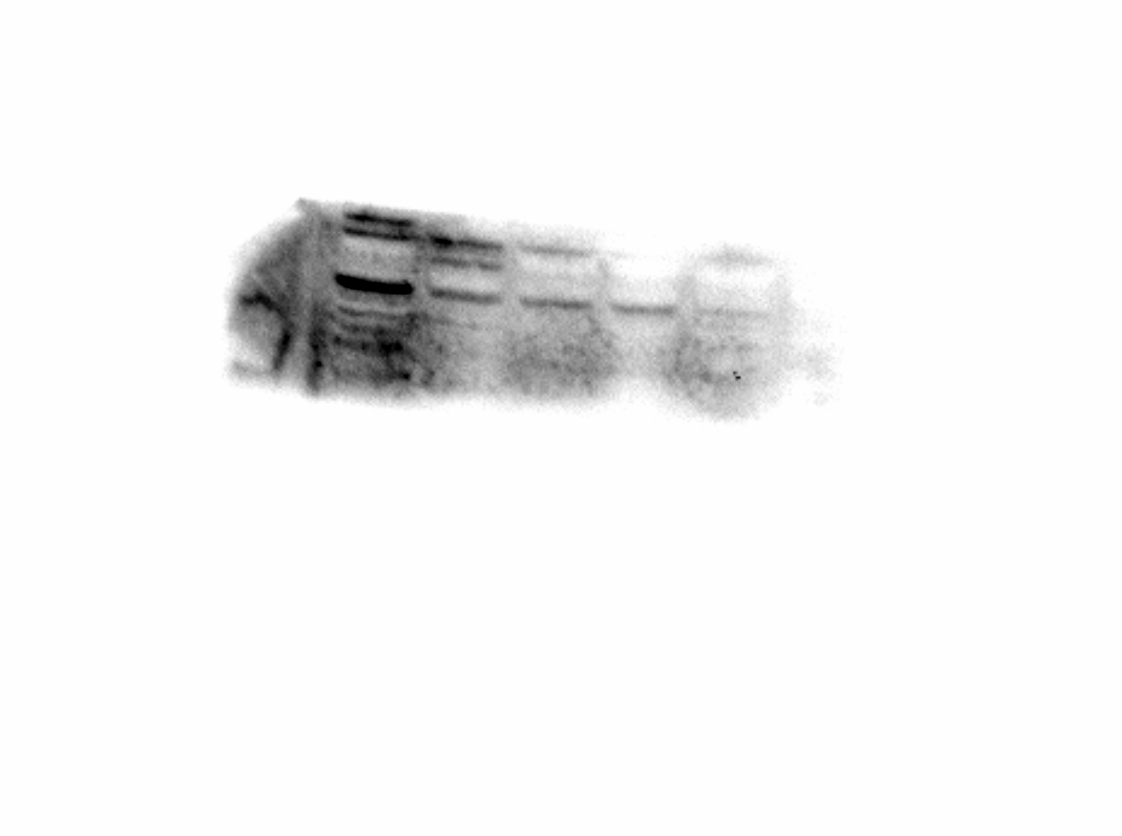

Supplement: Supplemental Information 1 [file peerj-12-18406-s001.zip › FOXD WB/FOXD1/FOXD(1).jpg]

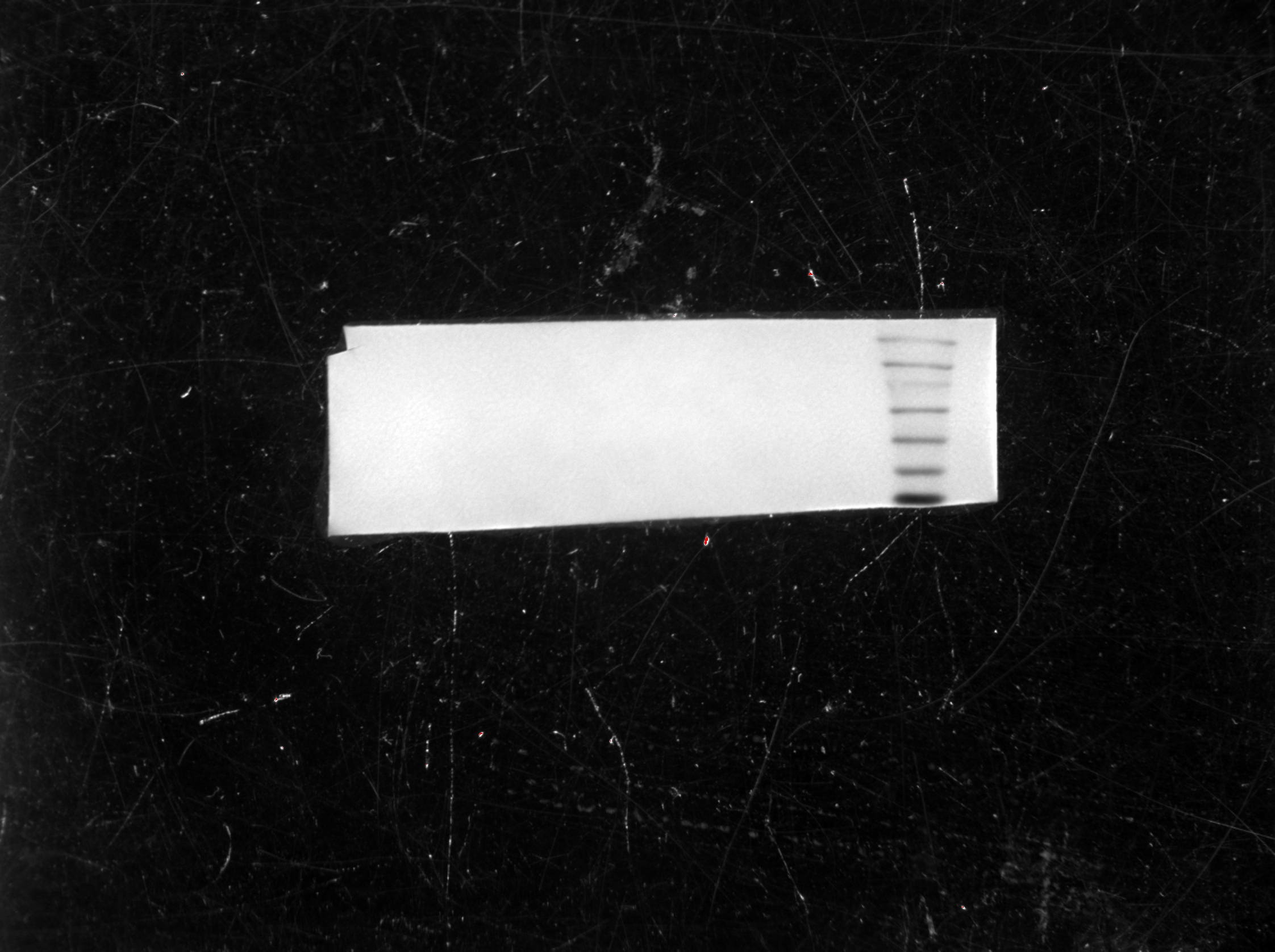

Supplement: Supplemental Information 1 [file peerj-12-18406-s001.zip › FOXD WB/FOXD1/FOXD1(1)marker.jpg]

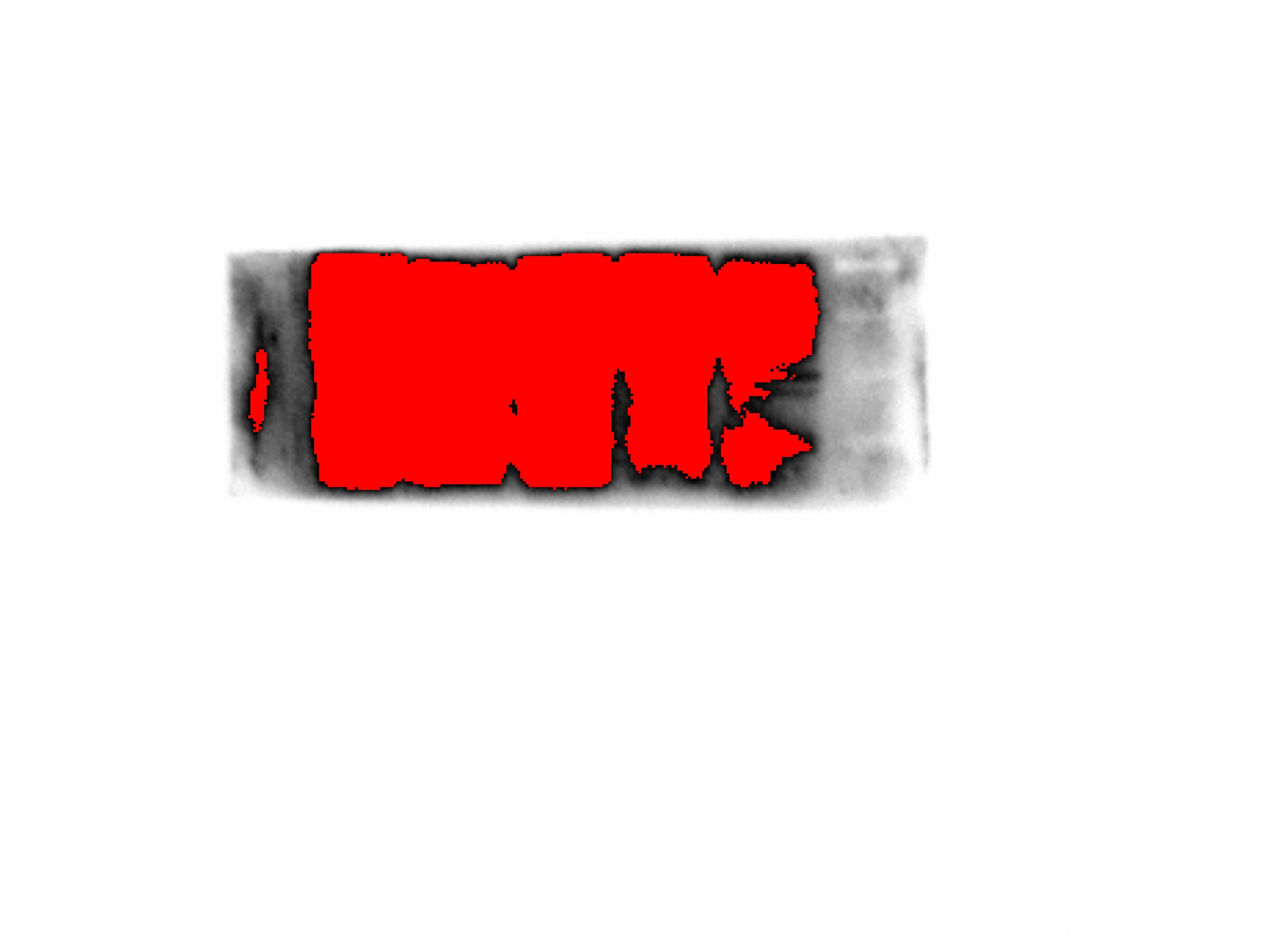

Supplement: Supplemental Information 1 [file peerj-12-18406-s001.zip › FOXD WB/FOXD1/FOXD1(2) marker.jpg]

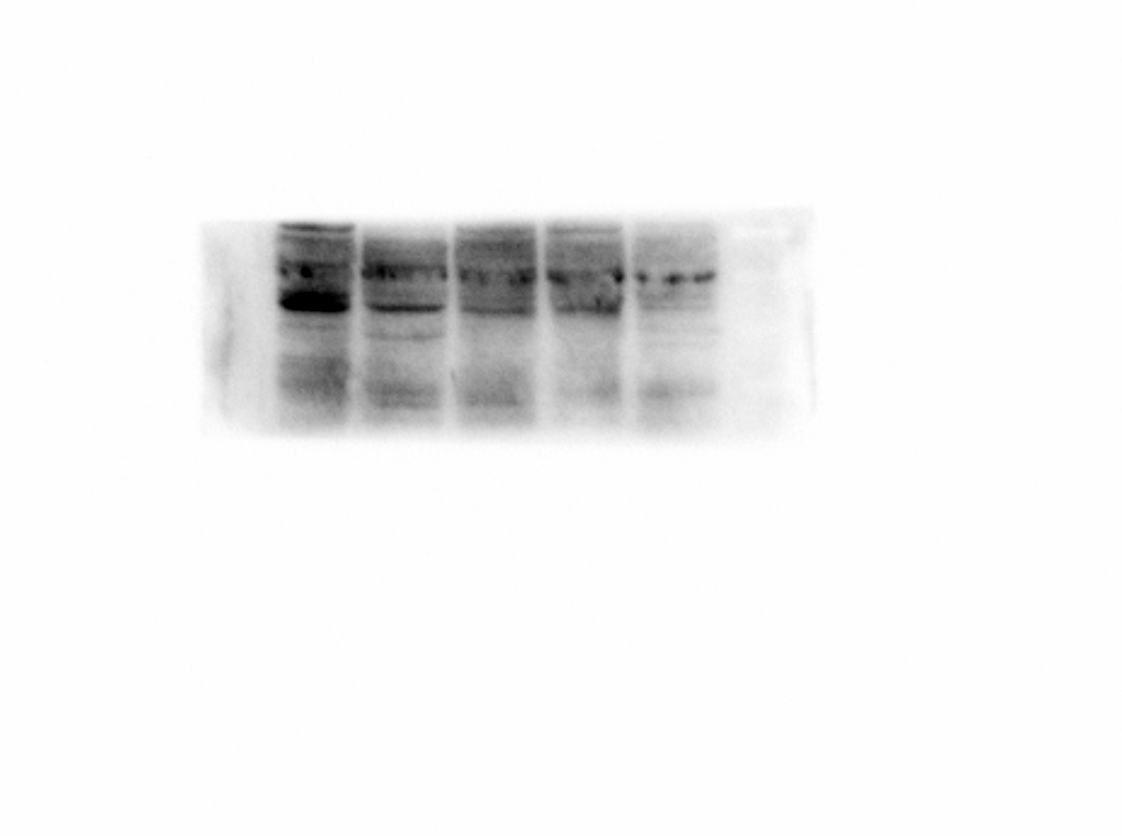

Supplement: Supplemental Information 1 [file peerj-12-18406-s001.zip › FOXD WB/FOXD1/FOXD1(2).jpg]

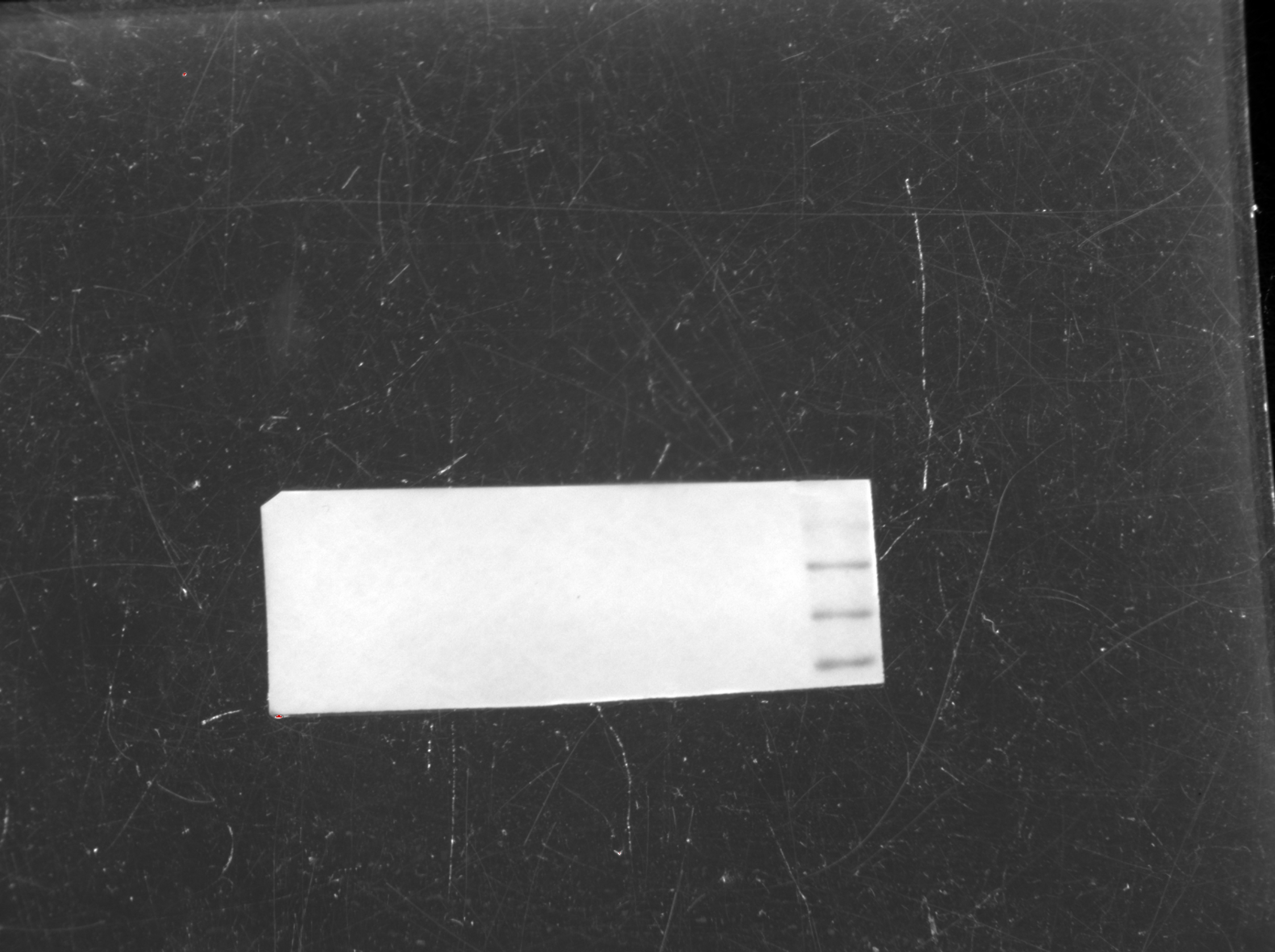

Supplement: Supplemental Information 1 [file peerj-12-18406-s001.zip › FOXD WB/FOXD1/FOXD1(3) marker.jpg]

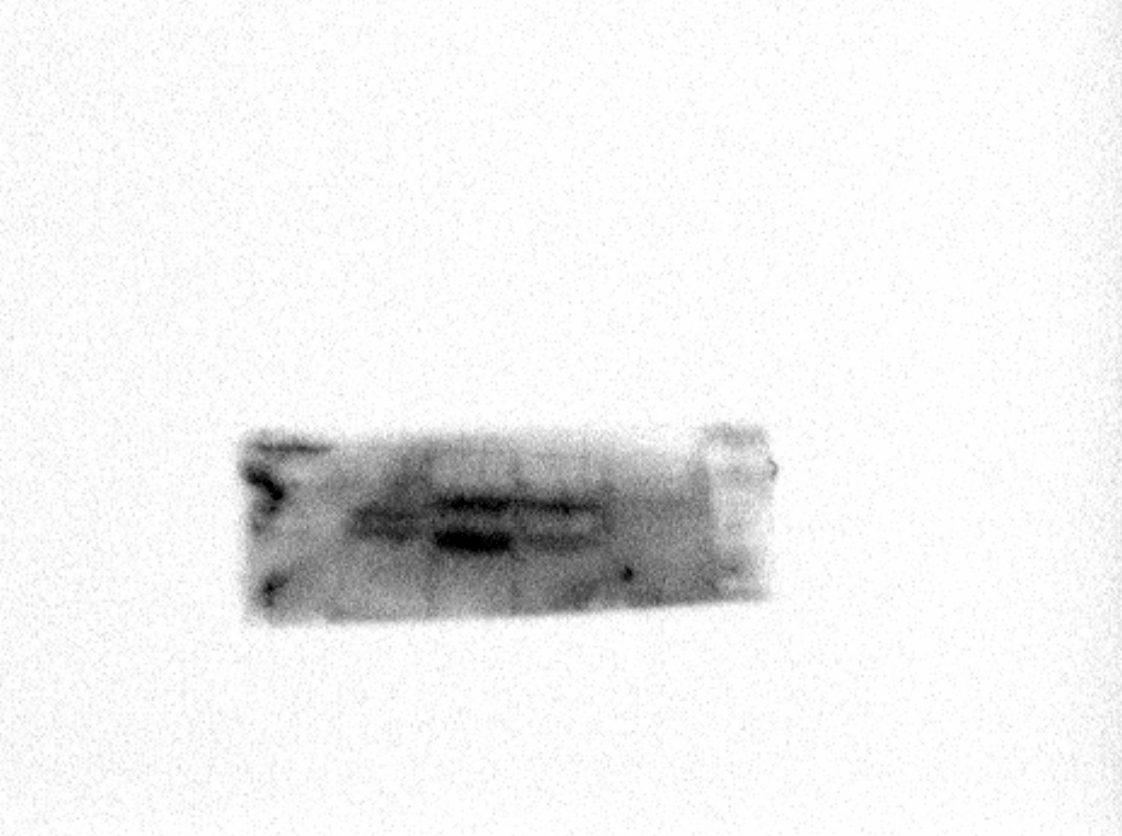

Supplement: Supplemental Information 1 [file peerj-12-18406-s001.zip › FOXD WB/FOXD1/FOXD1(3).jpg]

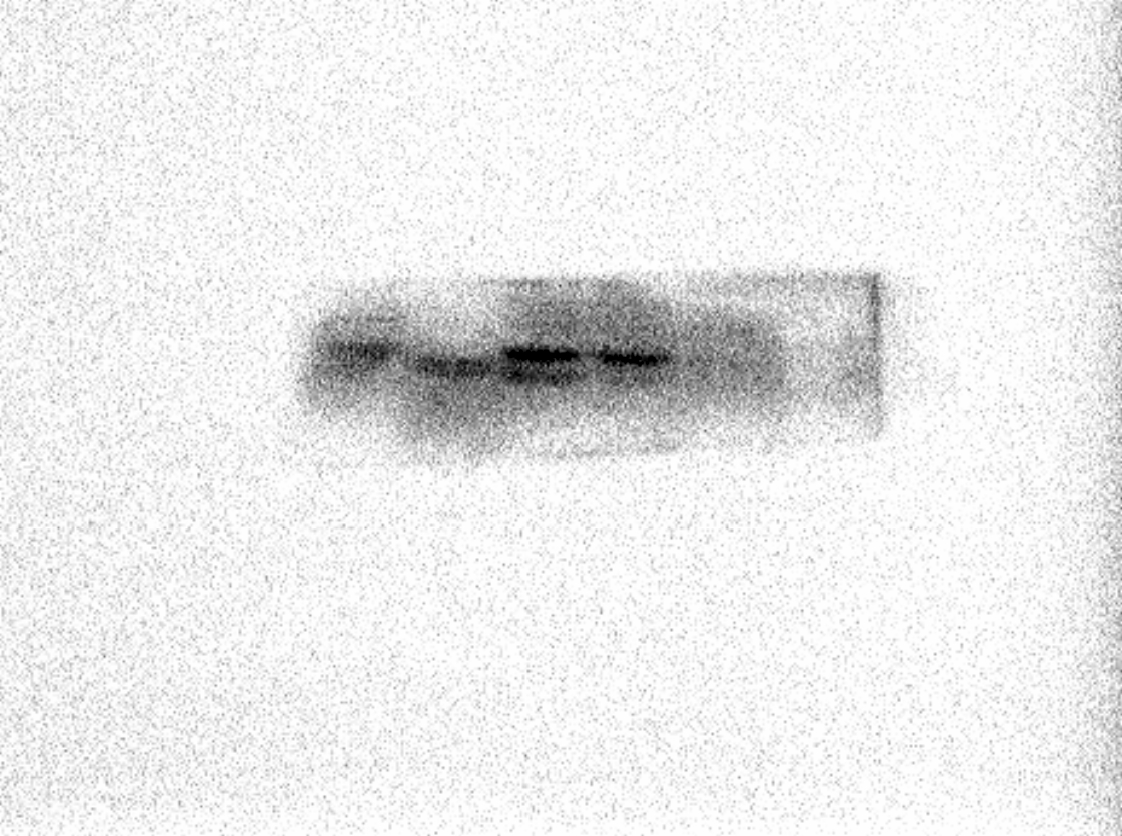

Supplement: Supplemental Information 1 [file peerj-12-18406-s001.zip › FOXD WB/FOXD1/FOXD1(4).jpg]

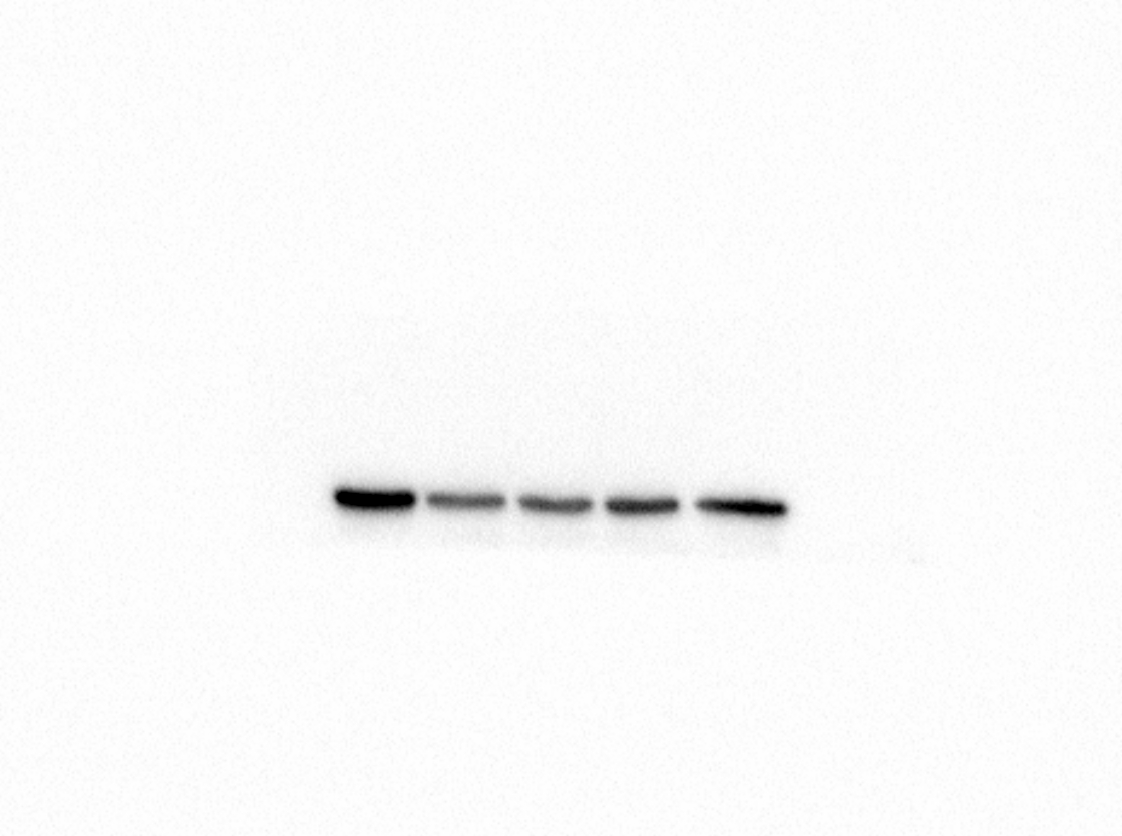

Supplement: Supplemental Information 1 [file peerj-12-18406-s001.zip › FOXD WB/FOXD1/GAPDH(1).jpg]

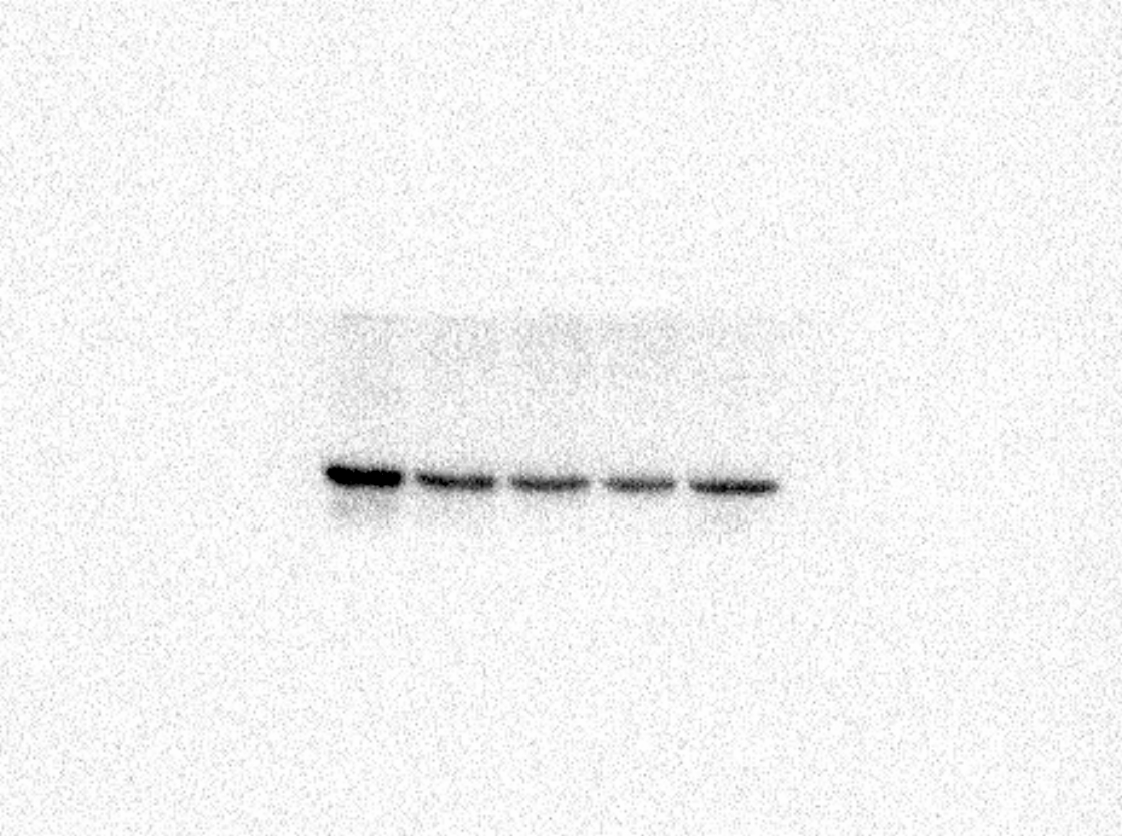

Supplement: Supplemental Information 1 [file peerj-12-18406-s001.zip › FOXD WB/FOXD1/GAPDH(2).jpg]

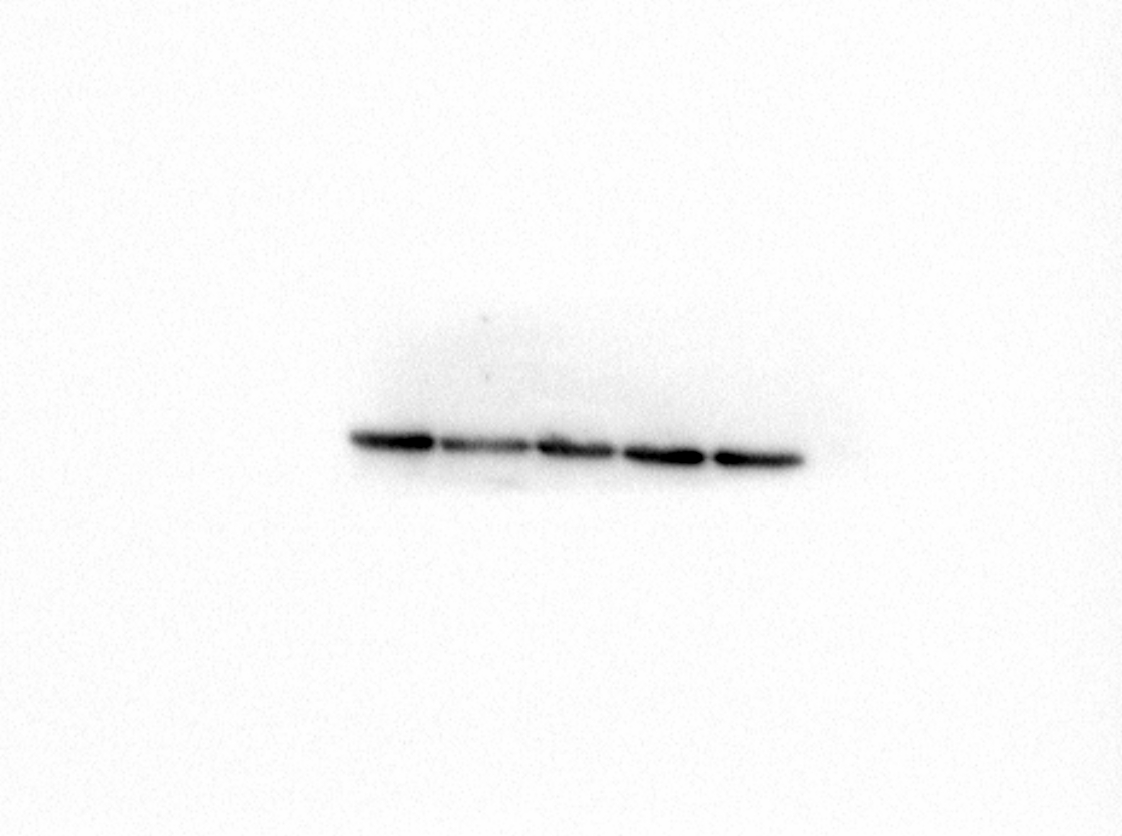

Supplement: Supplemental Information 1 [file peerj-12-18406-s001.zip › FOXD WB/FOXD1/GAPDH(3).jpg]

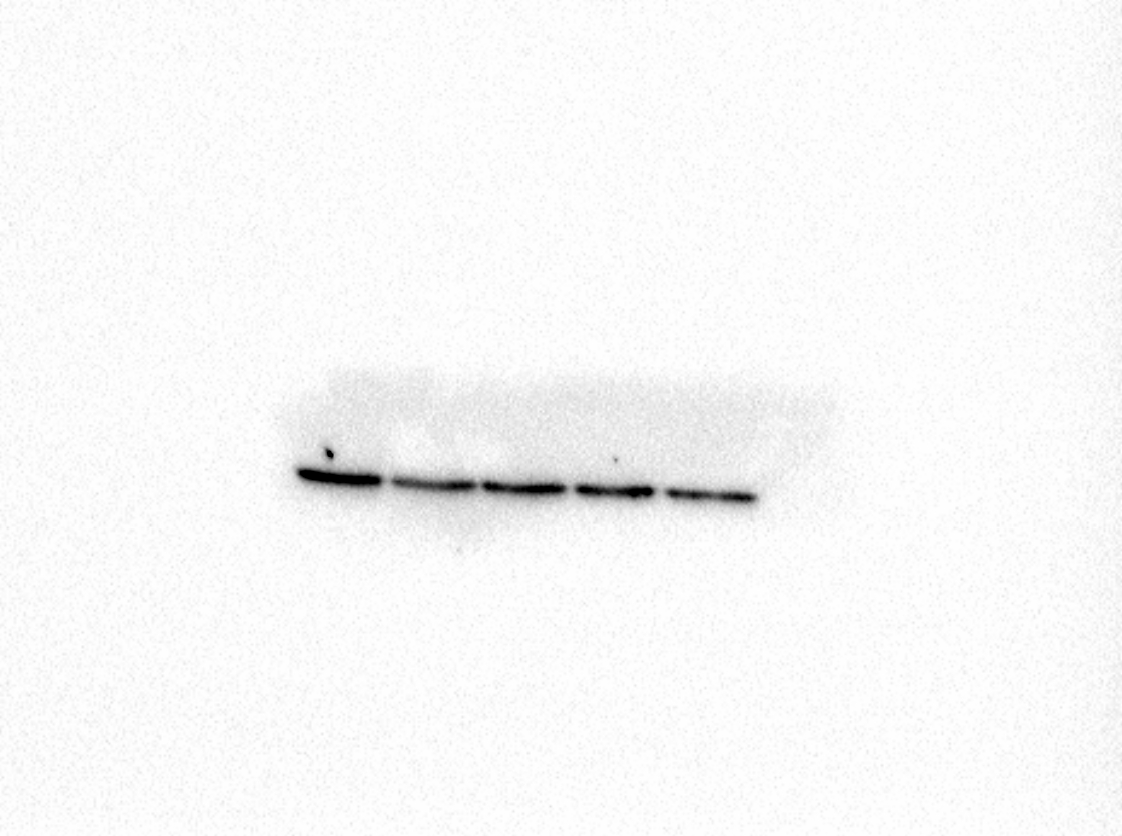

Supplement: Supplemental Information 1 [file peerj-12-18406-s001.zip › FOXD WB/FOXD1/GAPDH(4).jpg]

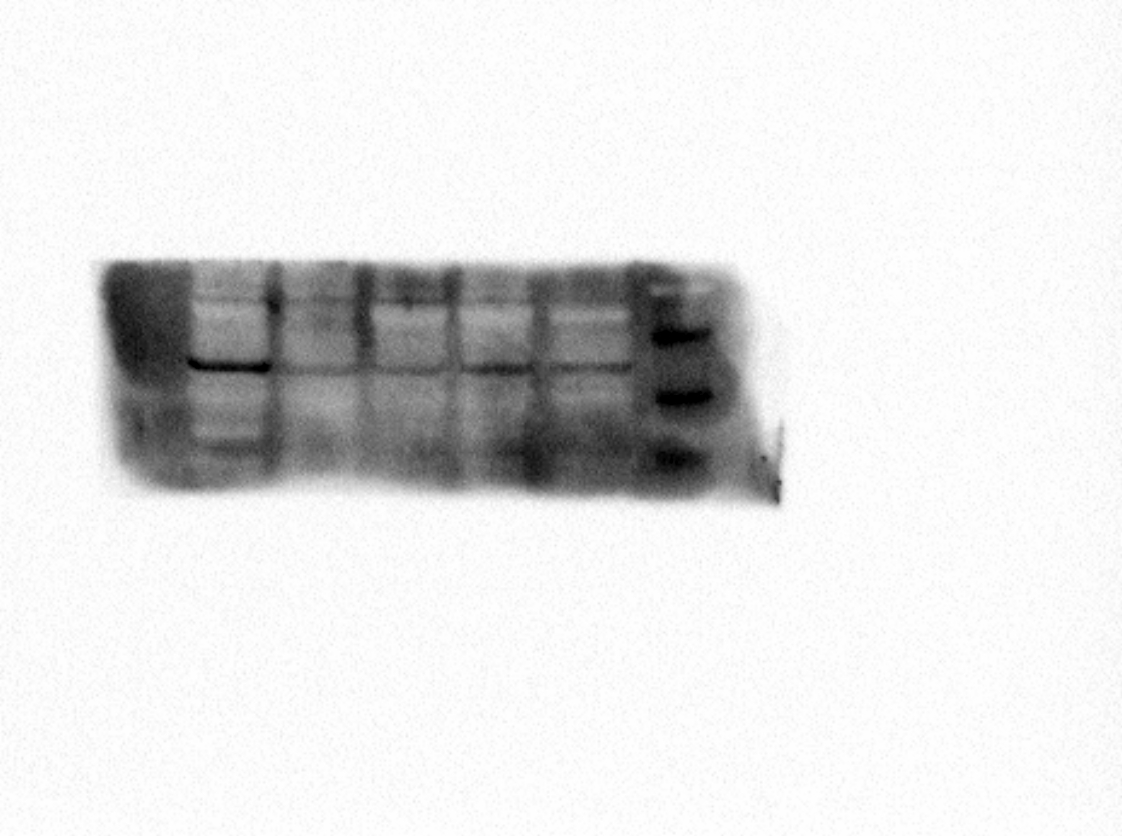

Supplement: Supplemental Information 1 [file peerj-12-18406-s001.zip › FOXD WB/FOXD2/FOXD2(1).jpg]

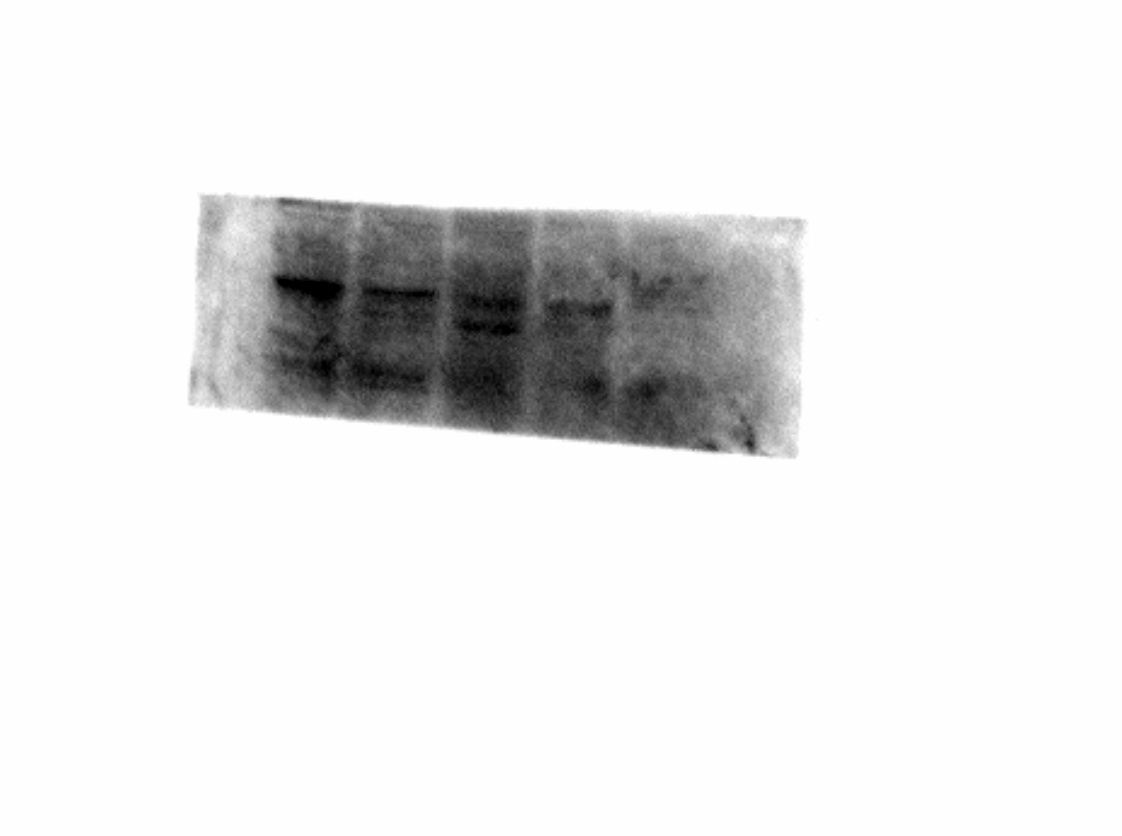

Supplement: Supplemental Information 1 [file peerj-12-18406-s001.zip › FOXD WB/FOXD2/FOXD2(2).jpg]

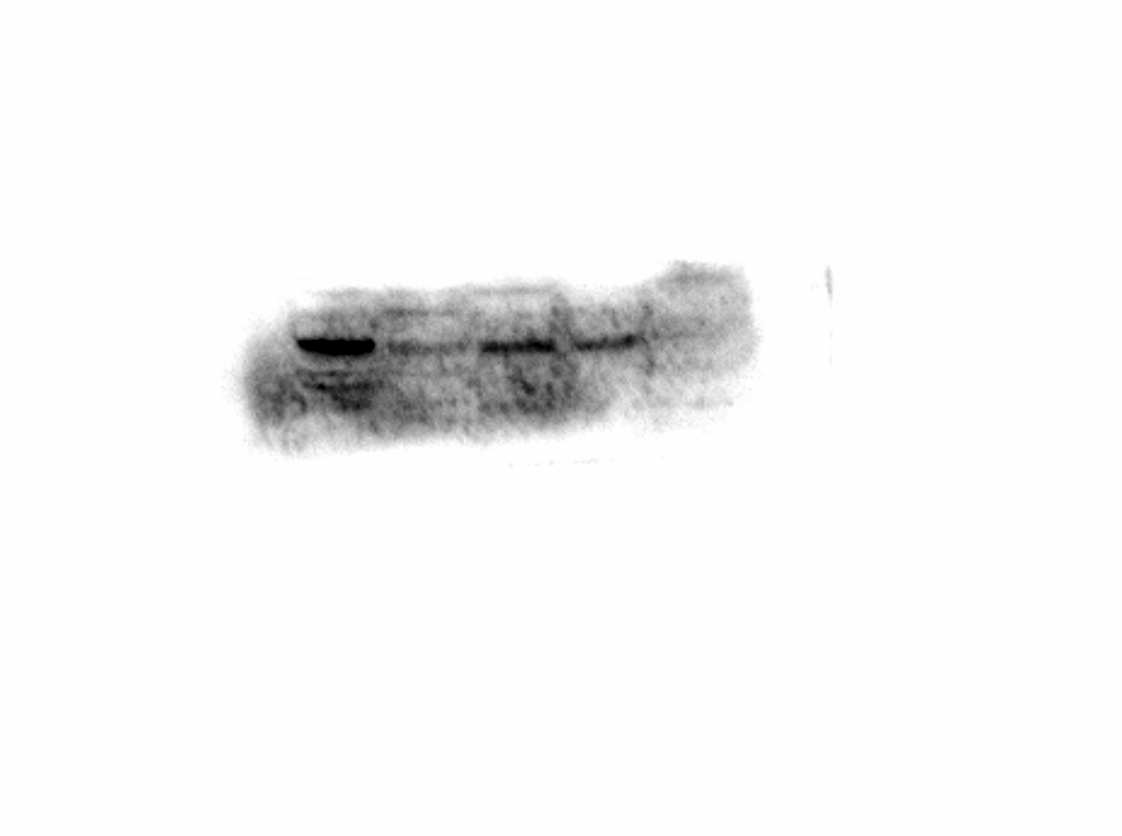

Supplement: Supplemental Information 1 [file peerj-12-18406-s001.zip › FOXD WB/FOXD2/FOXD2(3).jpg]

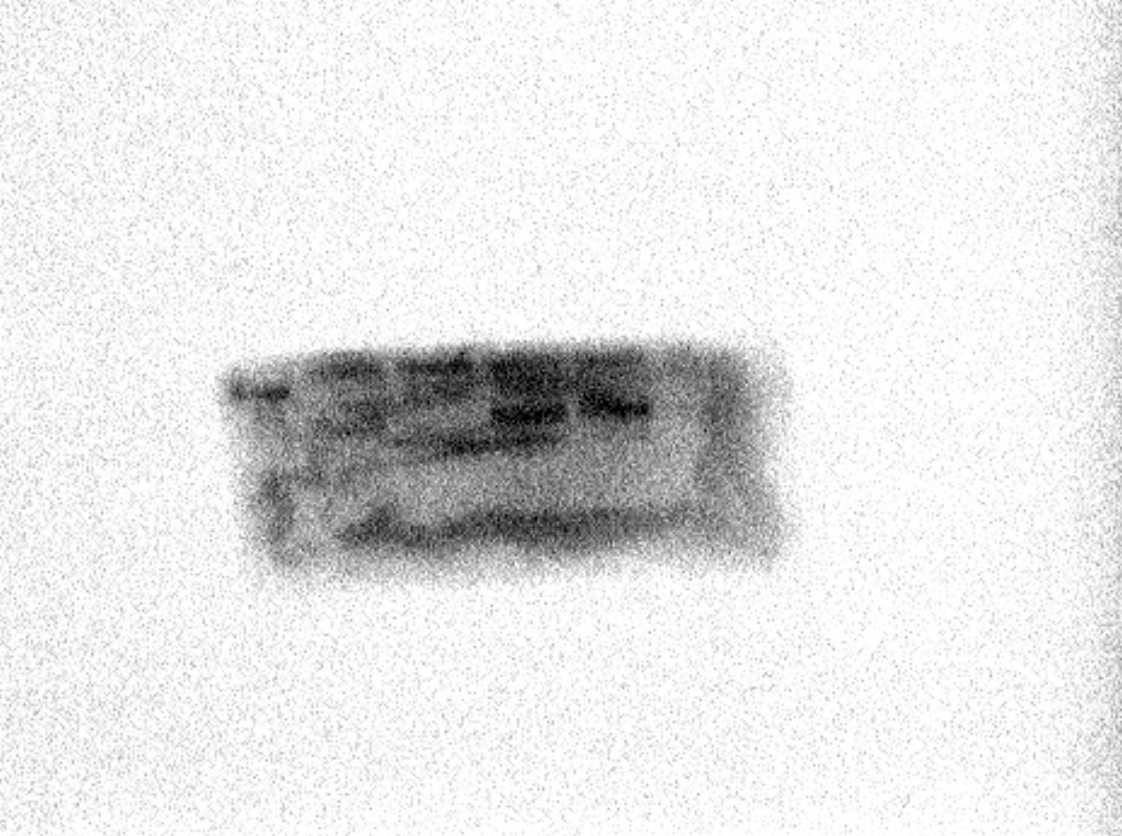

Supplement: Supplemental Information 1 [file peerj-12-18406-s001.zip › FOXD WB/FOXD2/FOXD2(4).jpg]

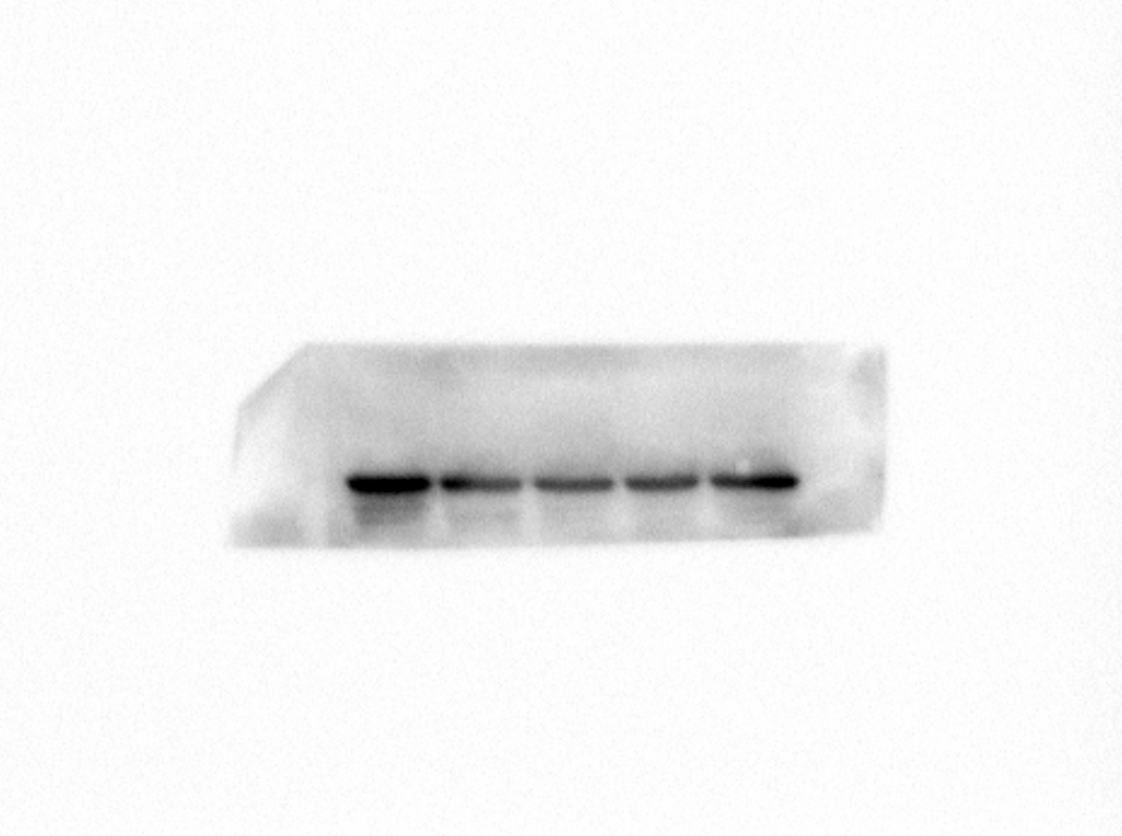

Supplement: Supplemental Information 1 [file peerj-12-18406-s001.zip › FOXD WB/FOXD2/GAPDH2(3).jpg]

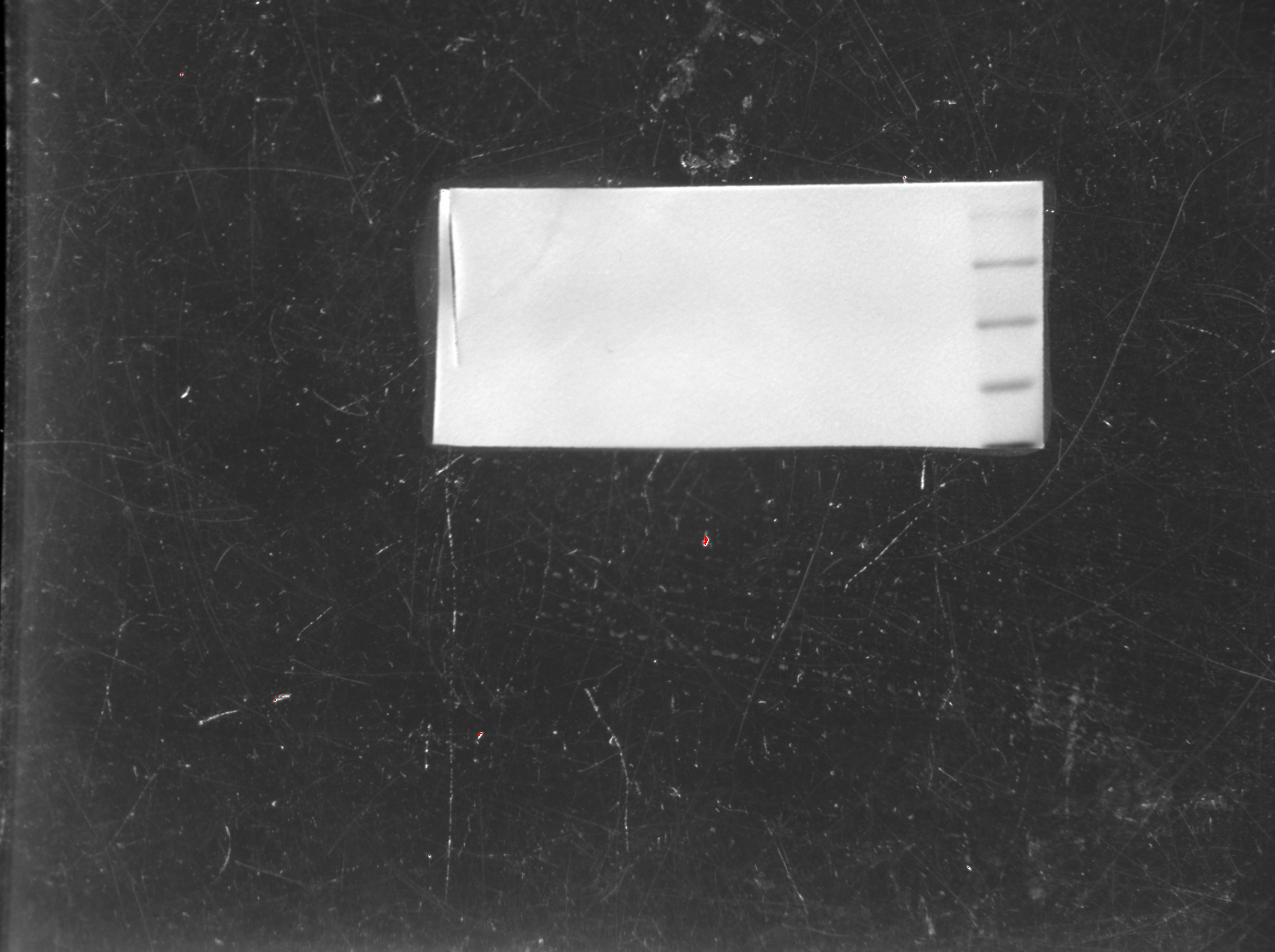

Supplement: Supplemental Information 1 [file peerj-12-18406-s001.zip › FOXD WB/FOXD3/FOXD3(5) marker.tif]

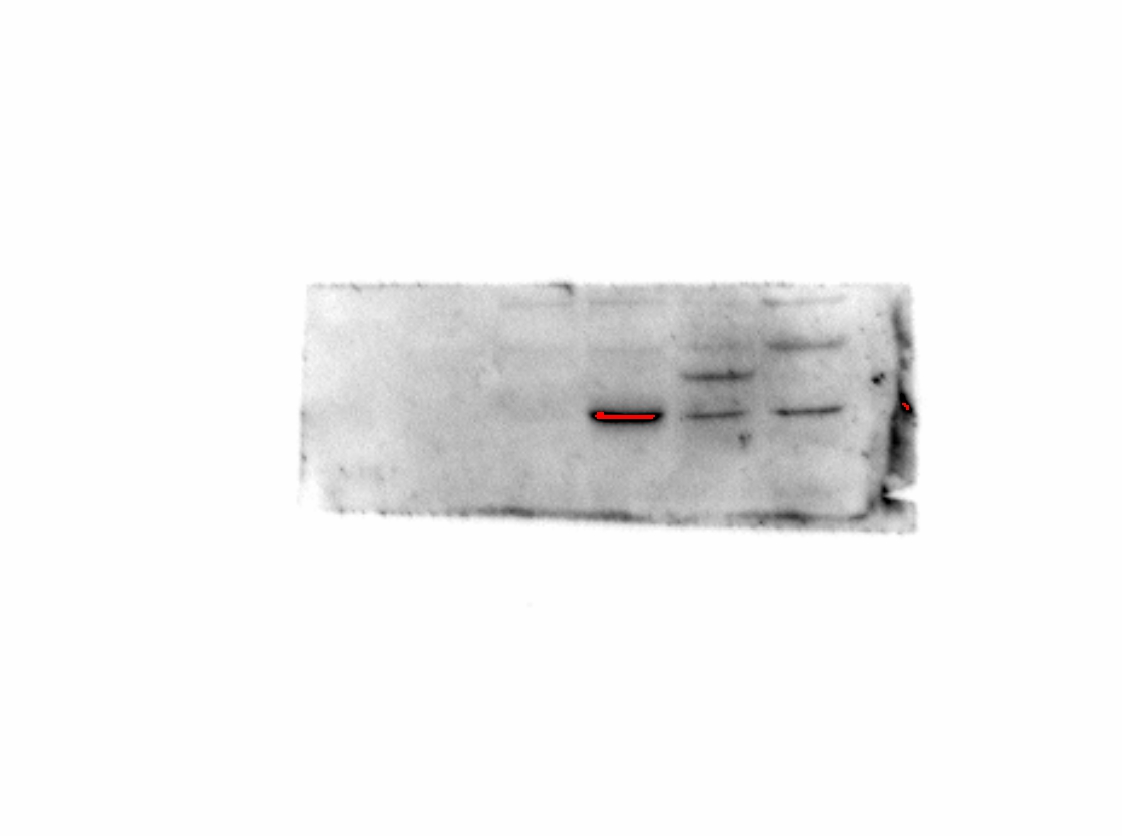

Supplement: Supplemental Information 1 [file peerj-12-18406-s001.zip › FOXD WB/FOXD3/FOXD3.1.jpg]

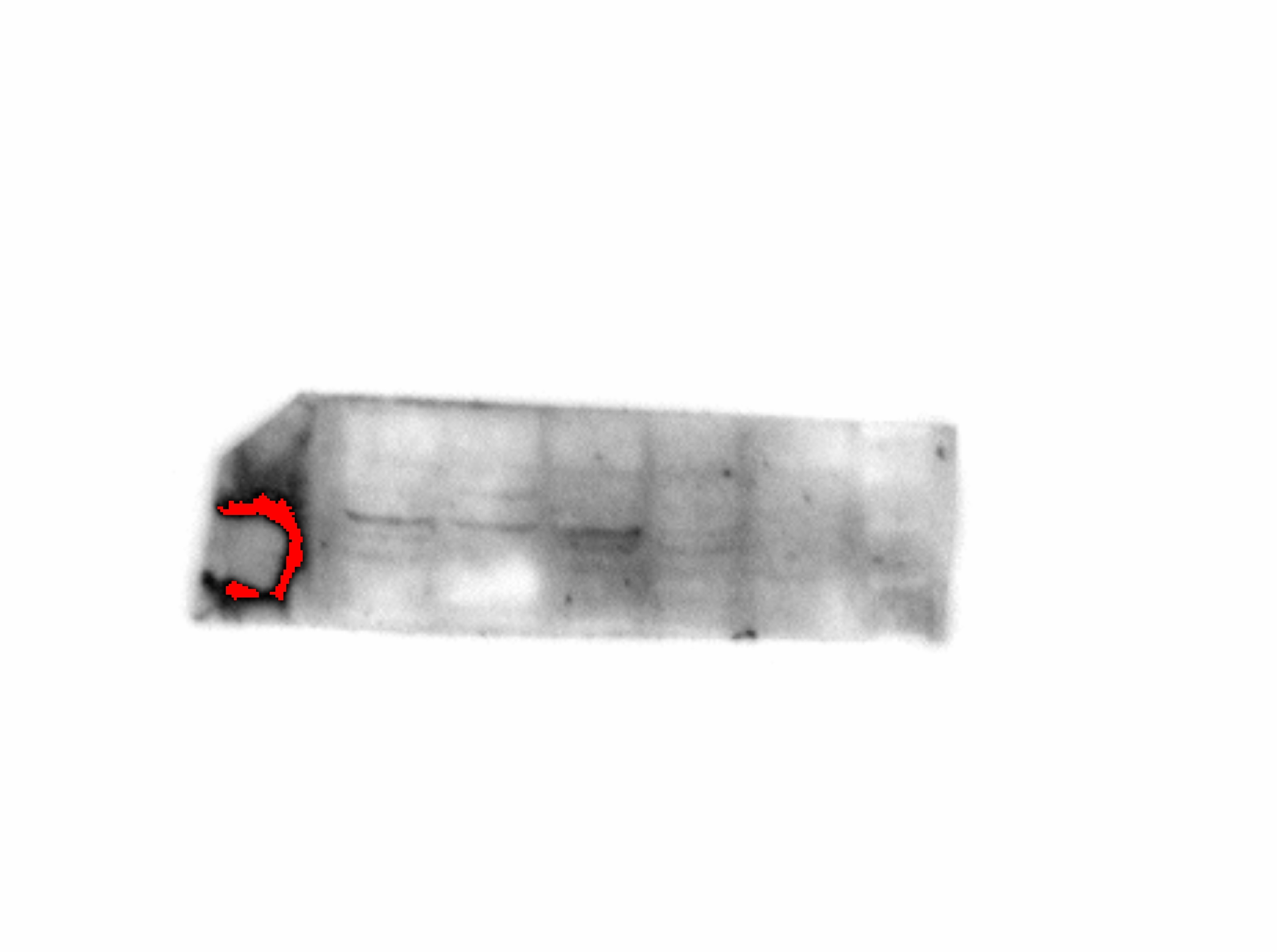

Supplement: Supplemental Information 1 [file peerj-12-18406-s001.zip › FOXD WB/FOXD3/FOXD3.2.jpg]

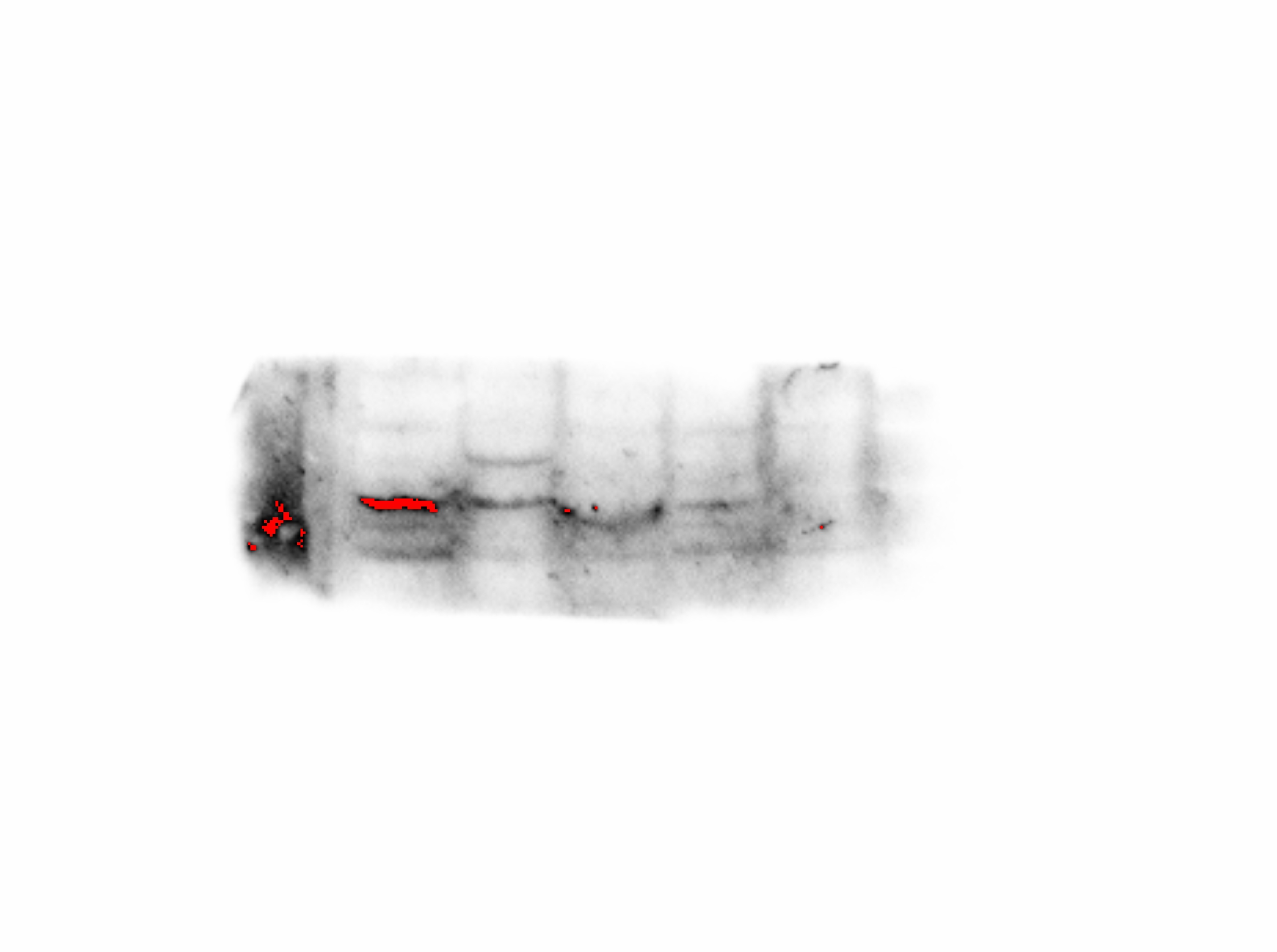

Supplement: Supplemental Information 1 [file peerj-12-18406-s001.zip › FOXD WB/FOXD3/FOXD3.3.jpg]

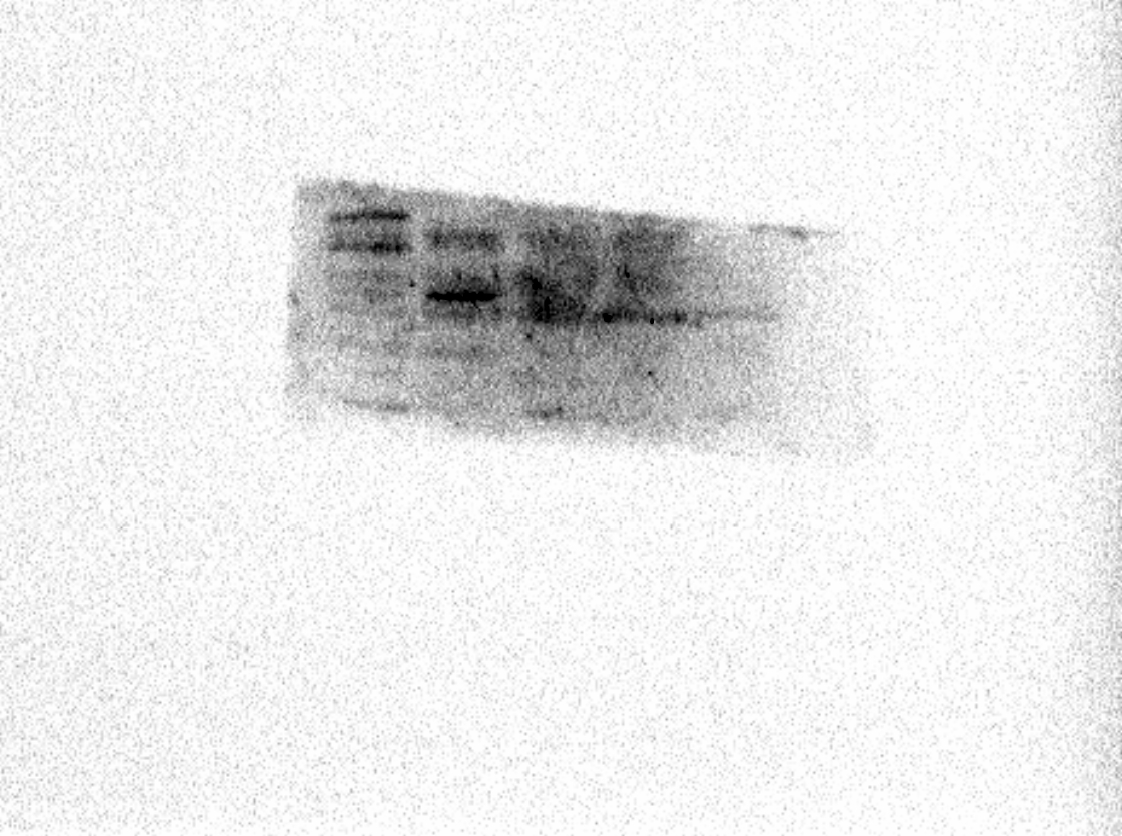

Supplement: Supplemental Information 1 [file peerj-12-18406-s001.zip › FOXD WB/FOXD3/FOXD3.4.jpg]

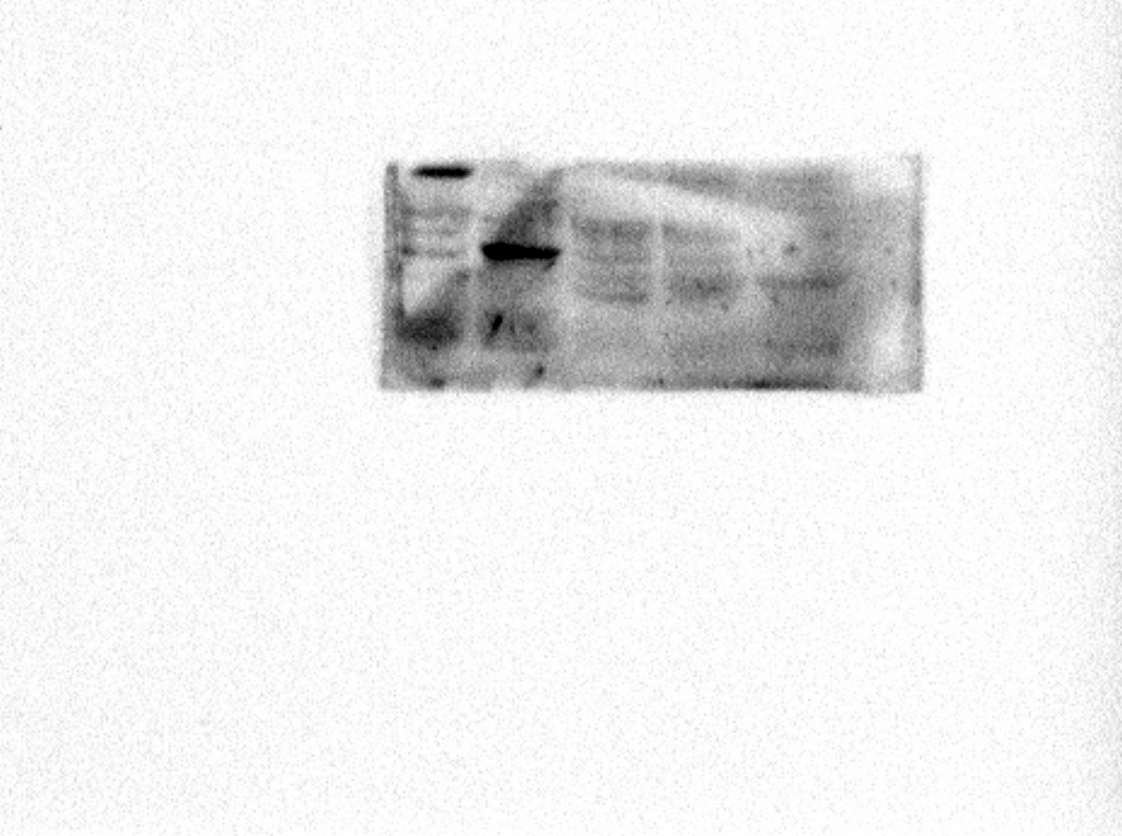

Supplement: Supplemental Information 1 [file peerj-12-18406-s001.zip › FOXD WB/FOXD3/FOXD3.5.jpg]

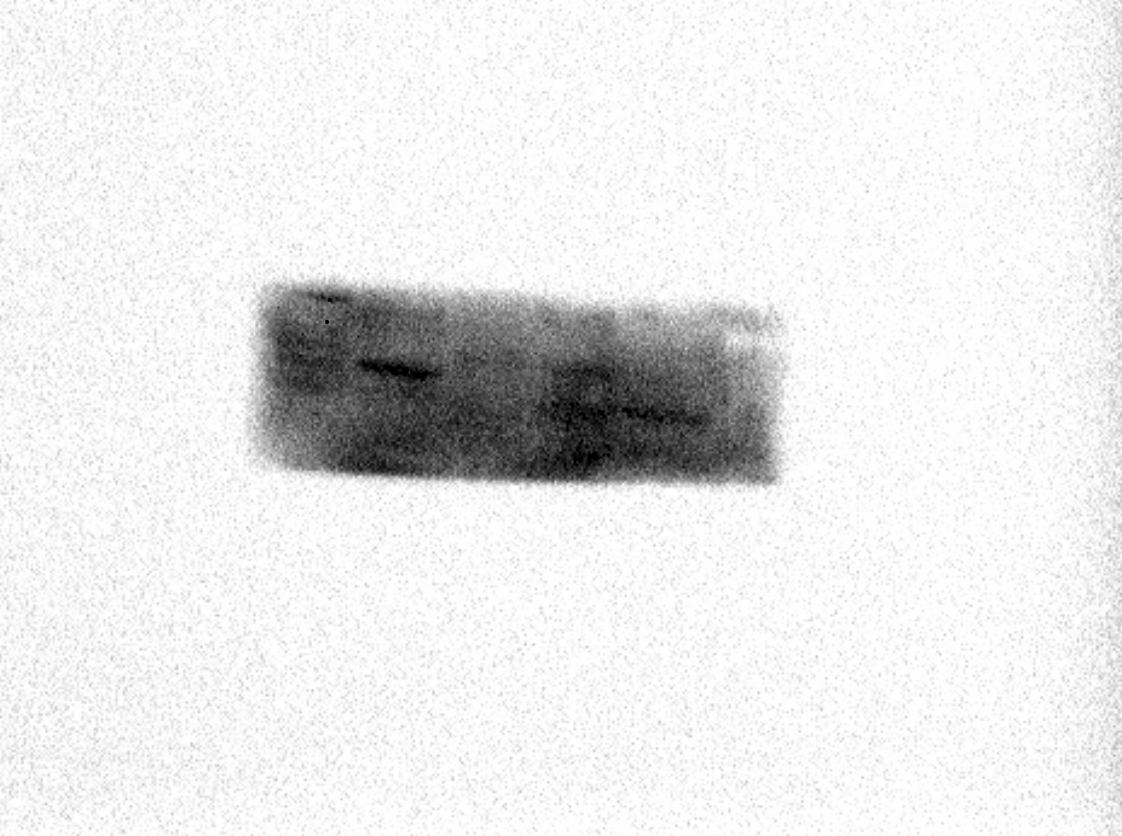

Supplement: Supplemental Information 1 [file peerj-12-18406-s001.zip › FOXD WB/FOXD3/FOXD3.6.jpg]

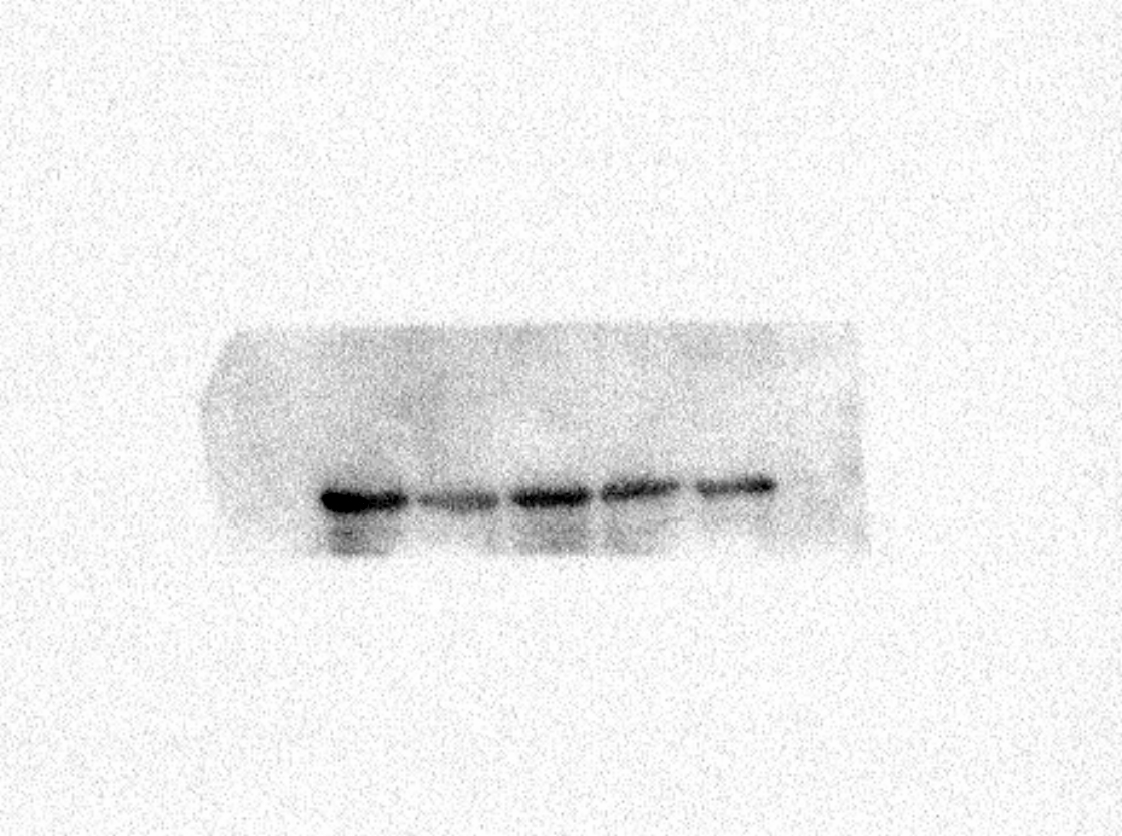

Supplement: Supplemental Information 1 [file peerj-12-18406-s001.zip › FOXD WB/FOXD3/GAPDH.3.jpg]

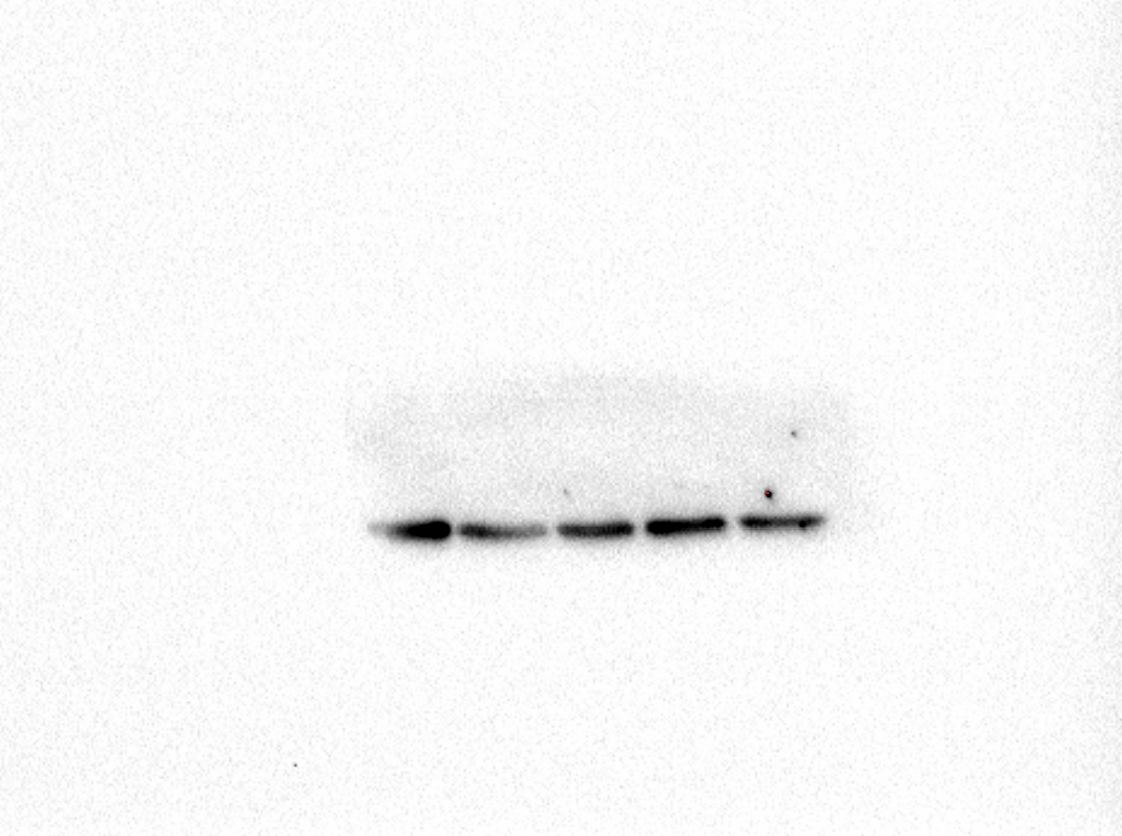

Supplement: Supplemental Information 1 [file peerj-12-18406-s001.zip › FOXD WB/FOXD3/gapdh.6.jpg]

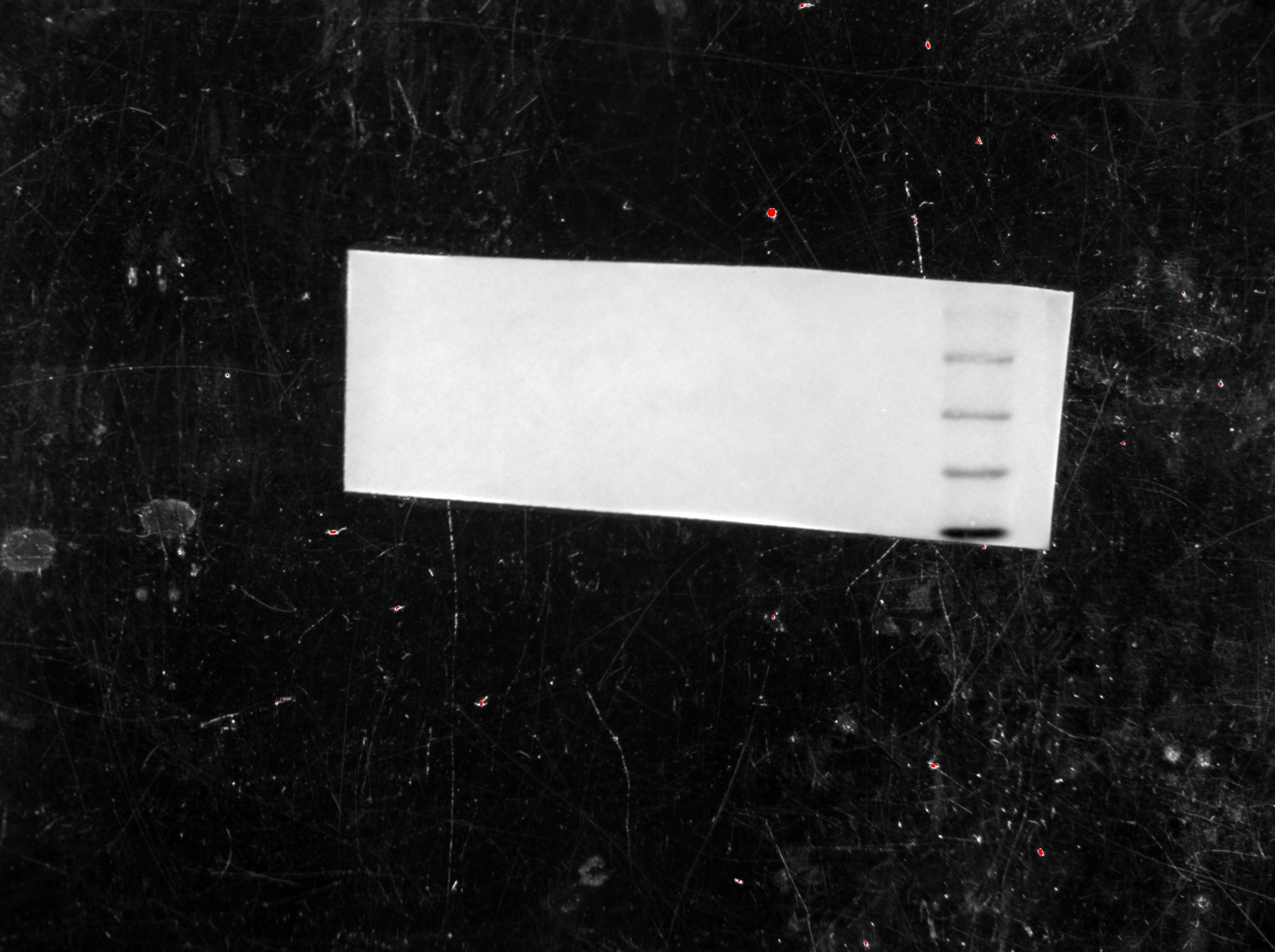

Supplement: Supplemental Information 1 [file peerj-12-18406-s001.zip › FOXD WB/FOXD4/FOXD4(3)marker.jpg]

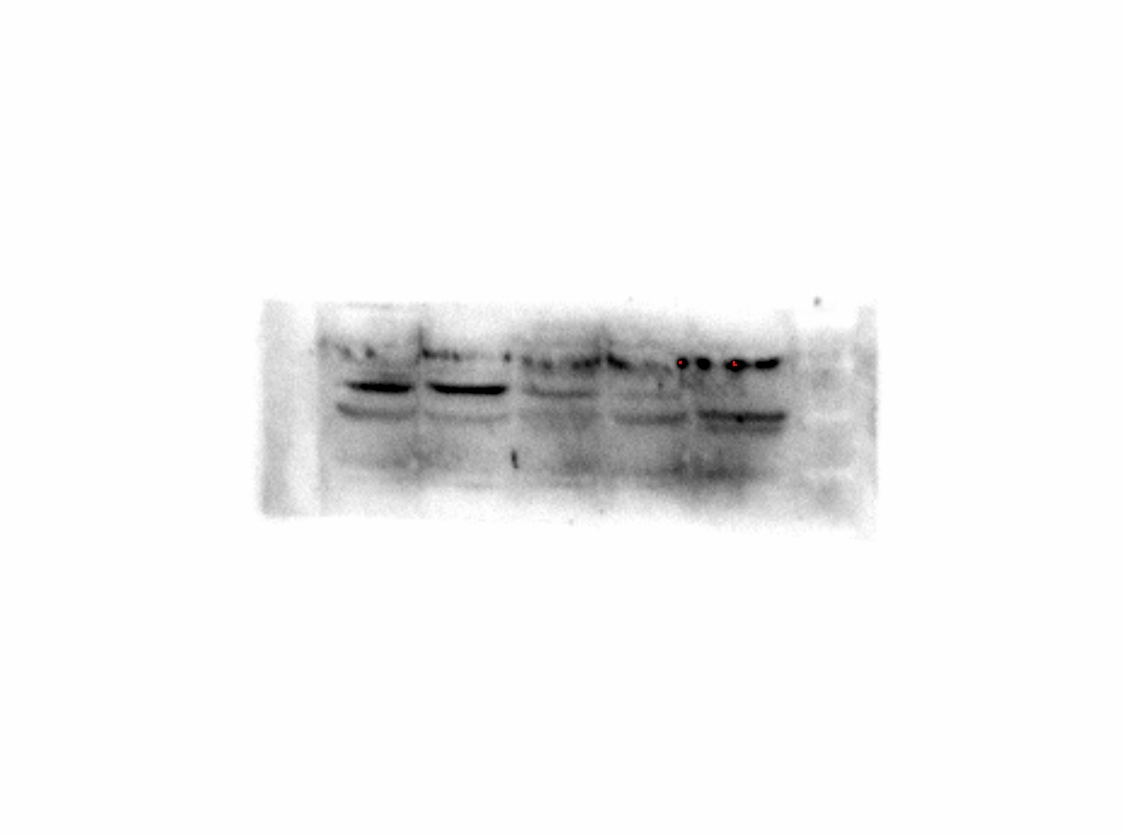

Supplement: Supplemental Information 1 [file peerj-12-18406-s001.zip › FOXD WB/FOXD4/FOXD4.1.jpg]

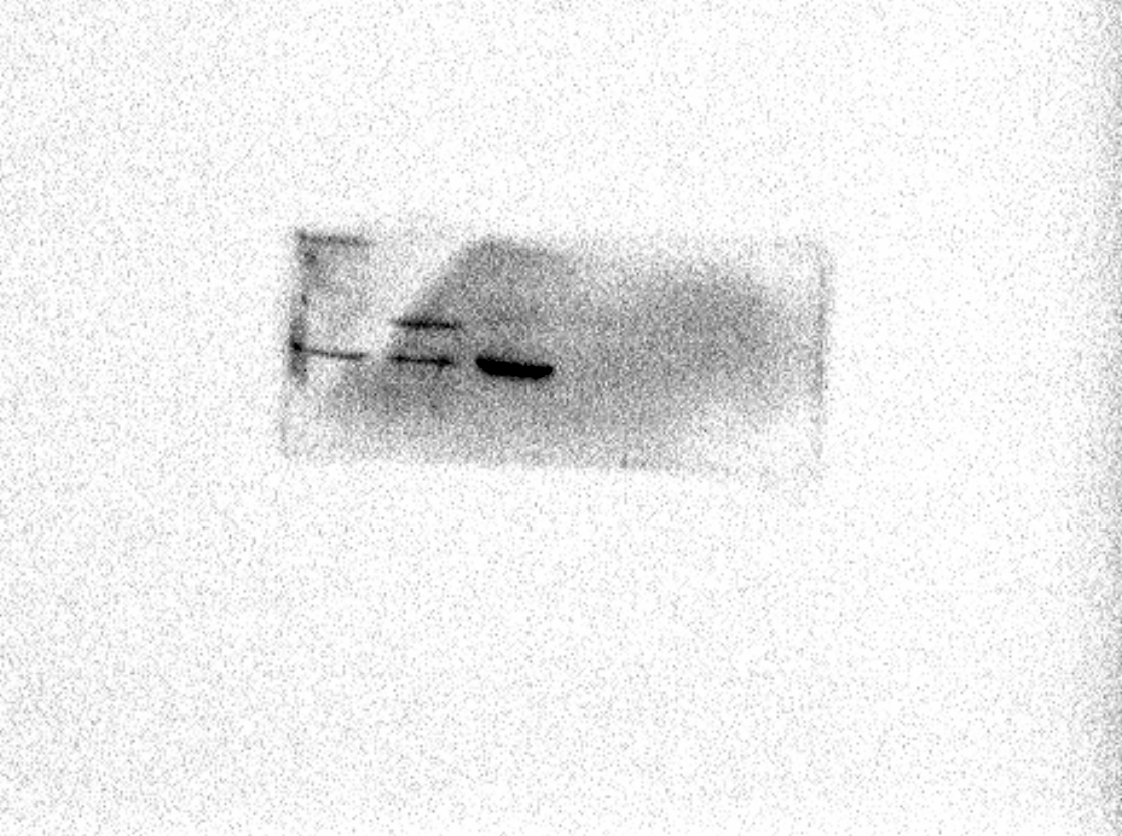

Supplement: Supplemental Information 1 [file peerj-12-18406-s001.zip › FOXD WB/FOXD4/FOXD4.2.jpg]

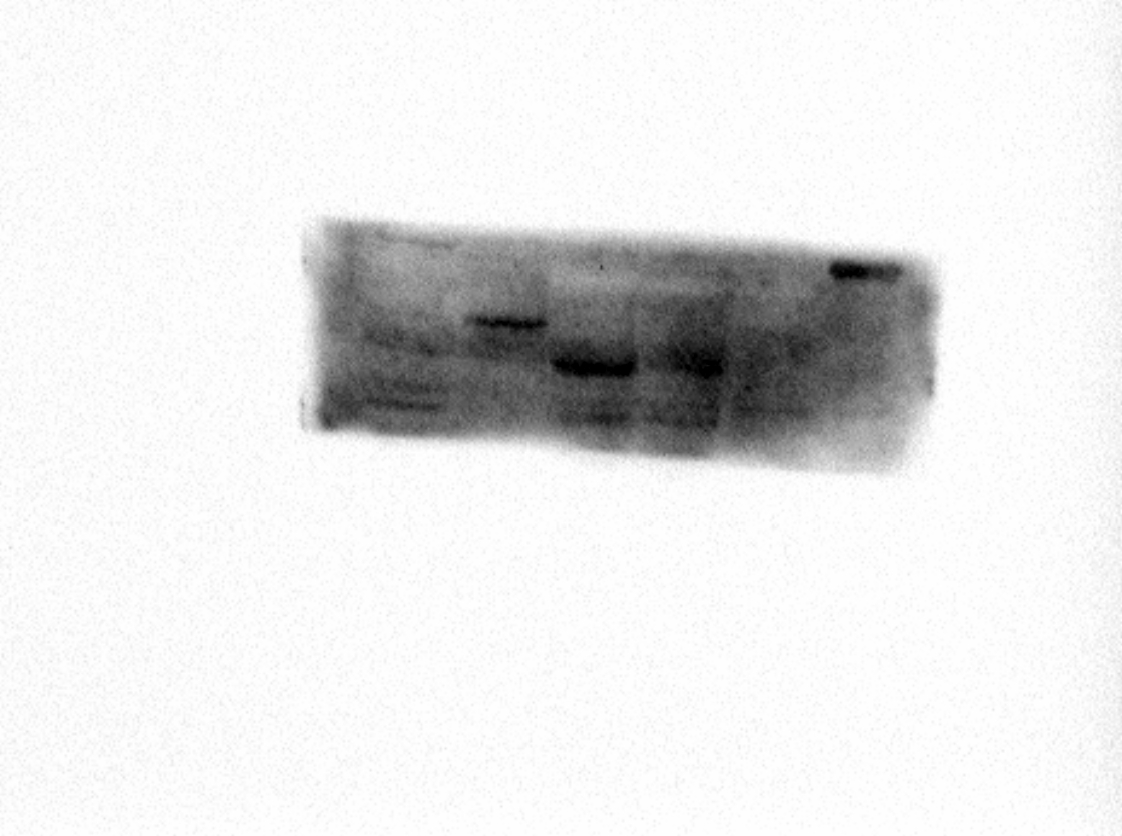

Supplement: Supplemental Information 1 [file peerj-12-18406-s001.zip › FOXD WB/FOXD4/FOXD4.3.jpg]

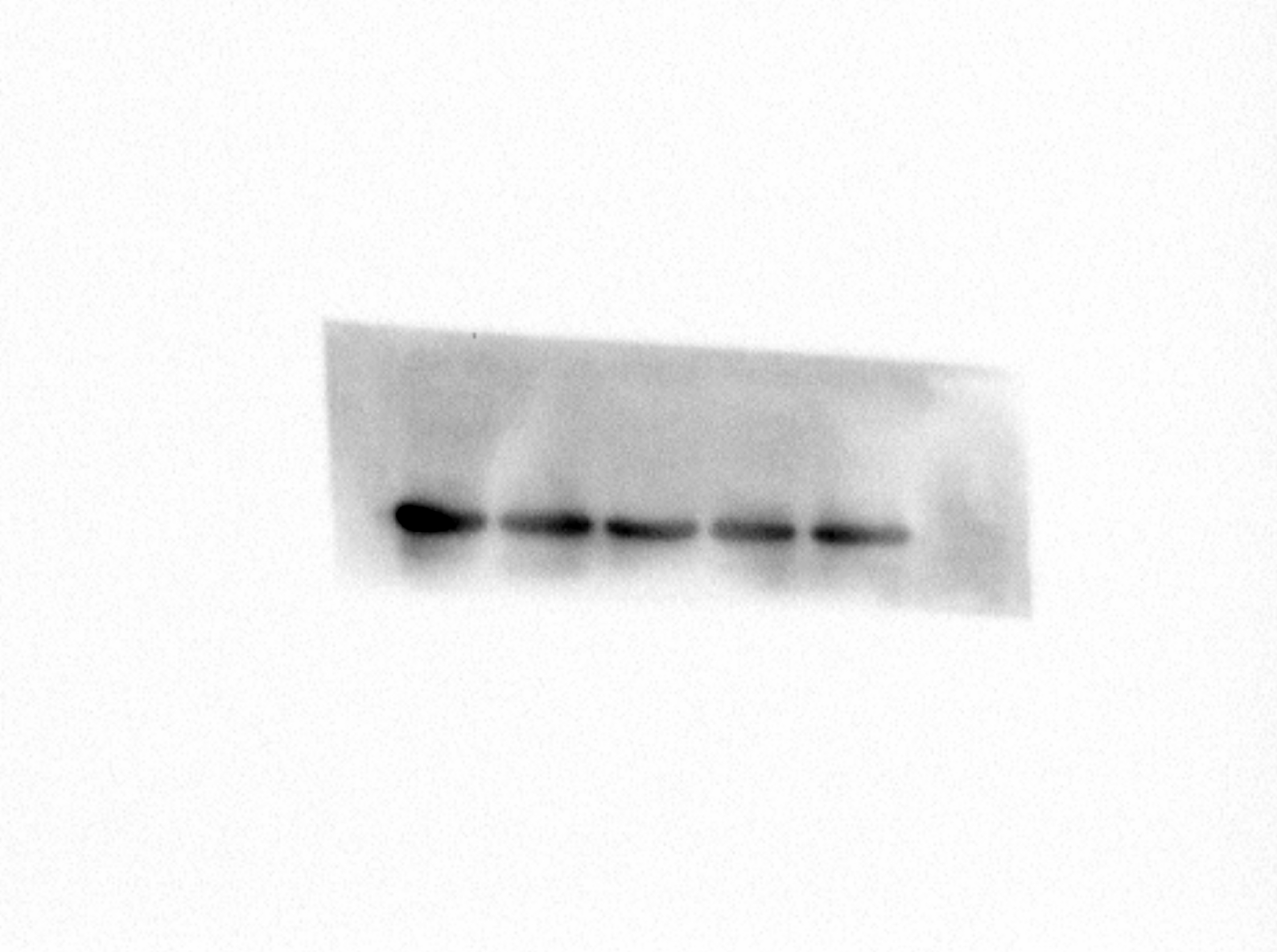

Supplement: Supplemental Information 1 [file peerj-12-18406-s001.zip › FOXD WB/FOXD4/GAPDH. 3.jpg]

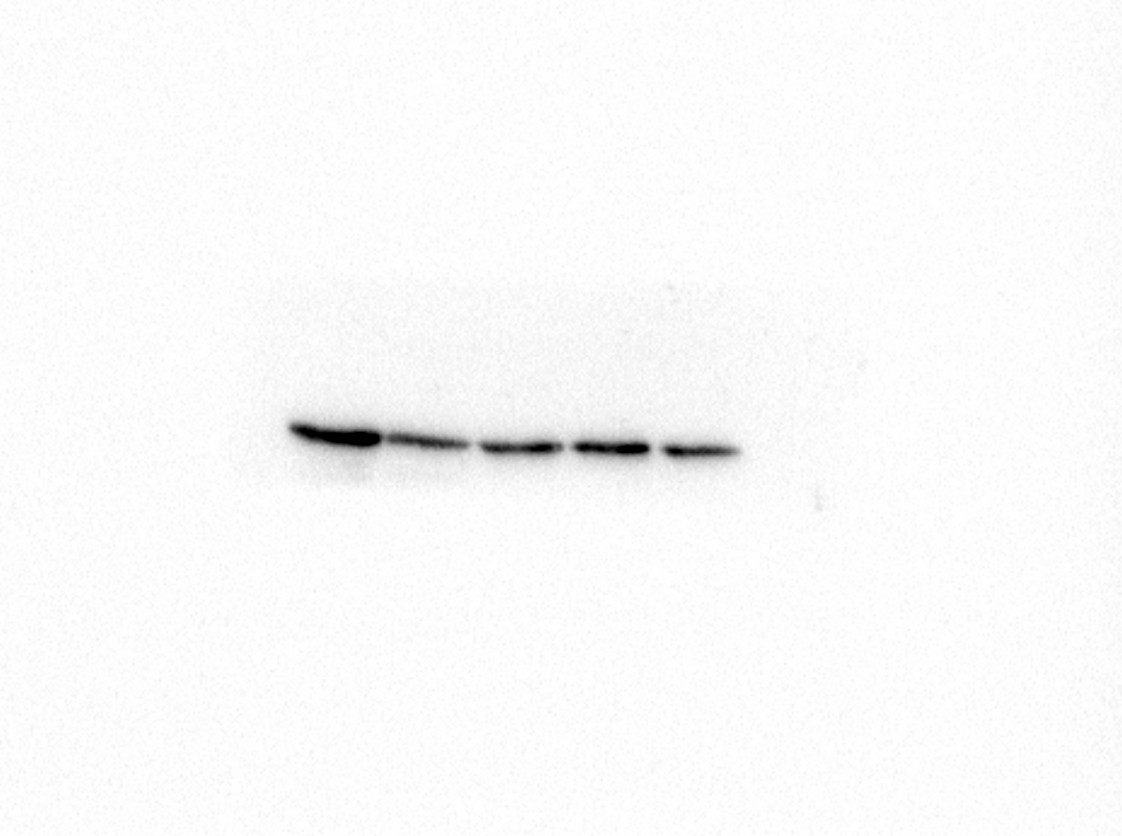

Supplement: Supplemental Information 1 [file peerj-12-18406-s001.zip › FOXD WB/FOXD4/GAPDH.2.jpg]

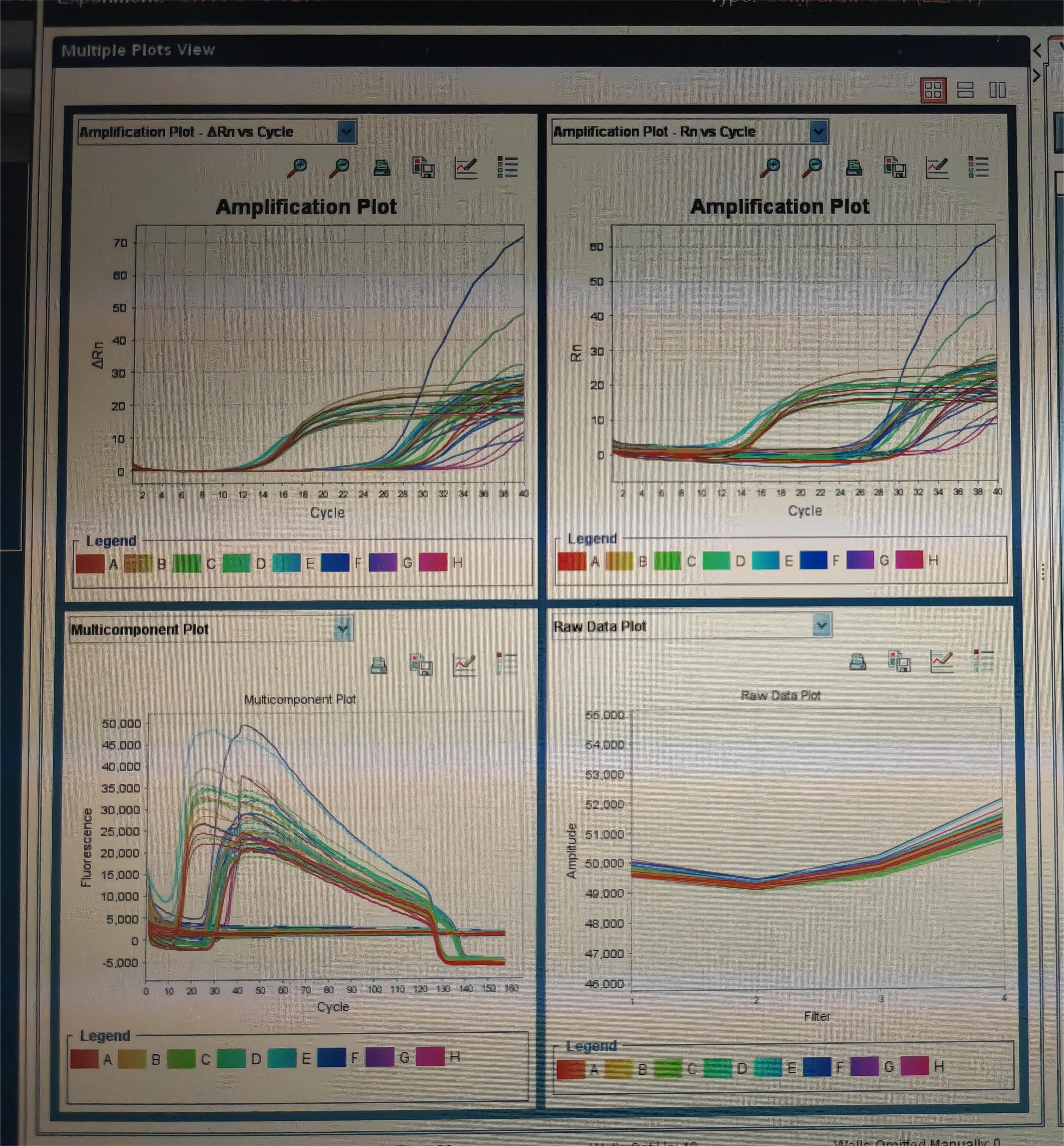

Supplement: Supplemental Information 2 [file peerj-12-18406-s002.zip › qPCR raw date/cell-1.jpg]

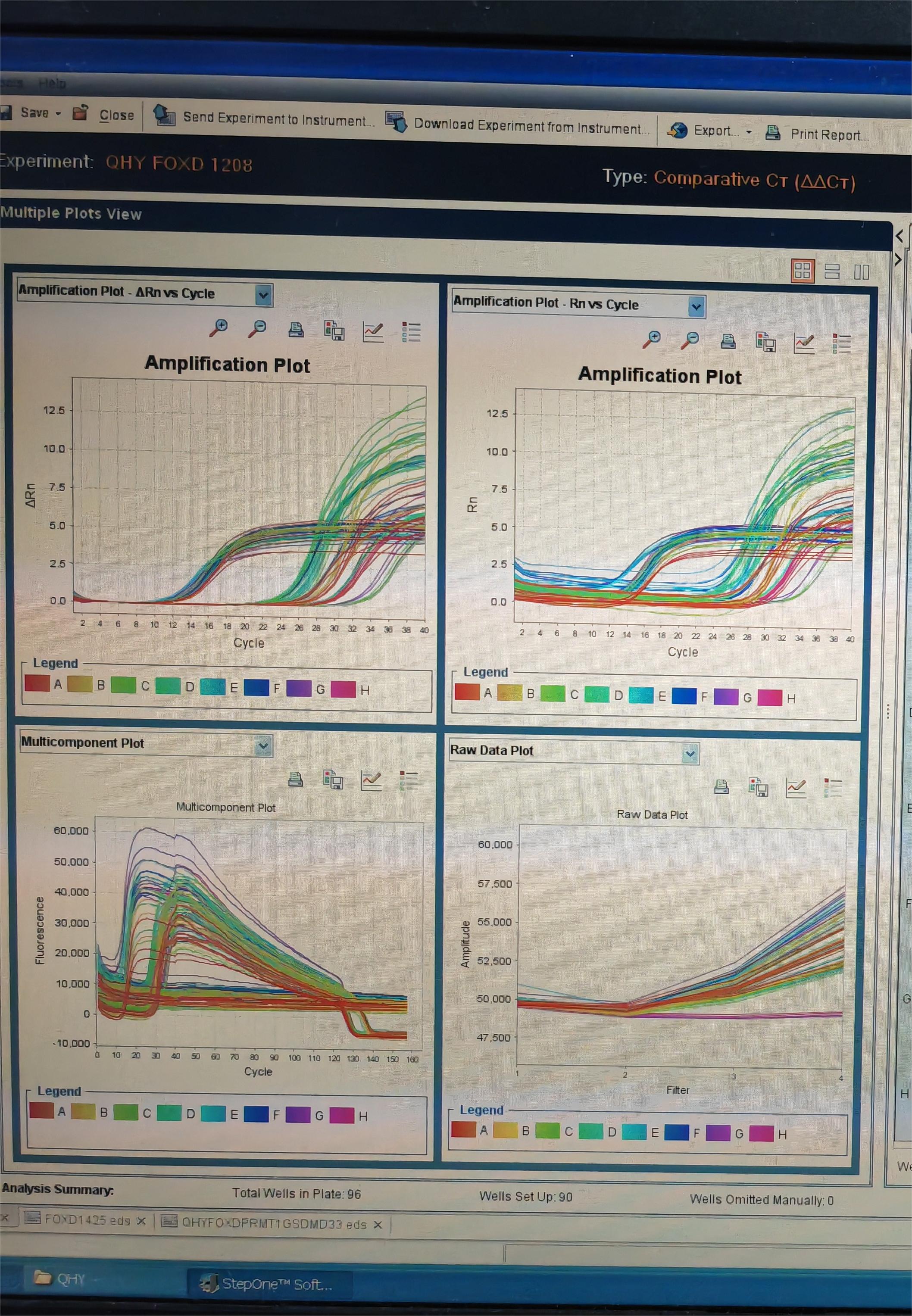

Supplement: Supplemental Information 2 [file peerj-12-18406-s002.zip › qPCR raw date/cell-2 cell-3.jpg]

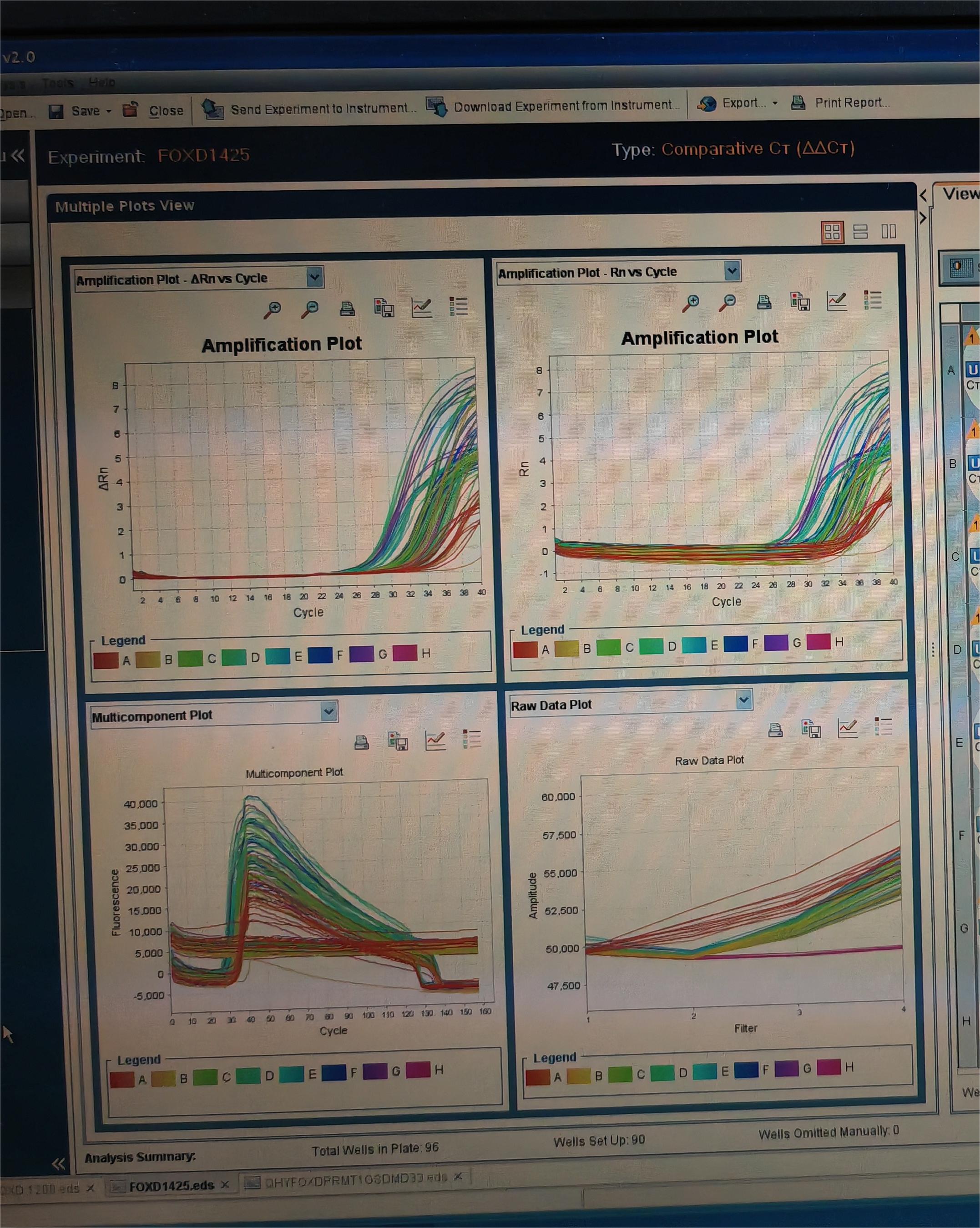

Supplement: Supplemental Information 2 [file peerj-12-18406-s002.zip › qPCR raw date/organization FOXD1 FOXD4.jpg]

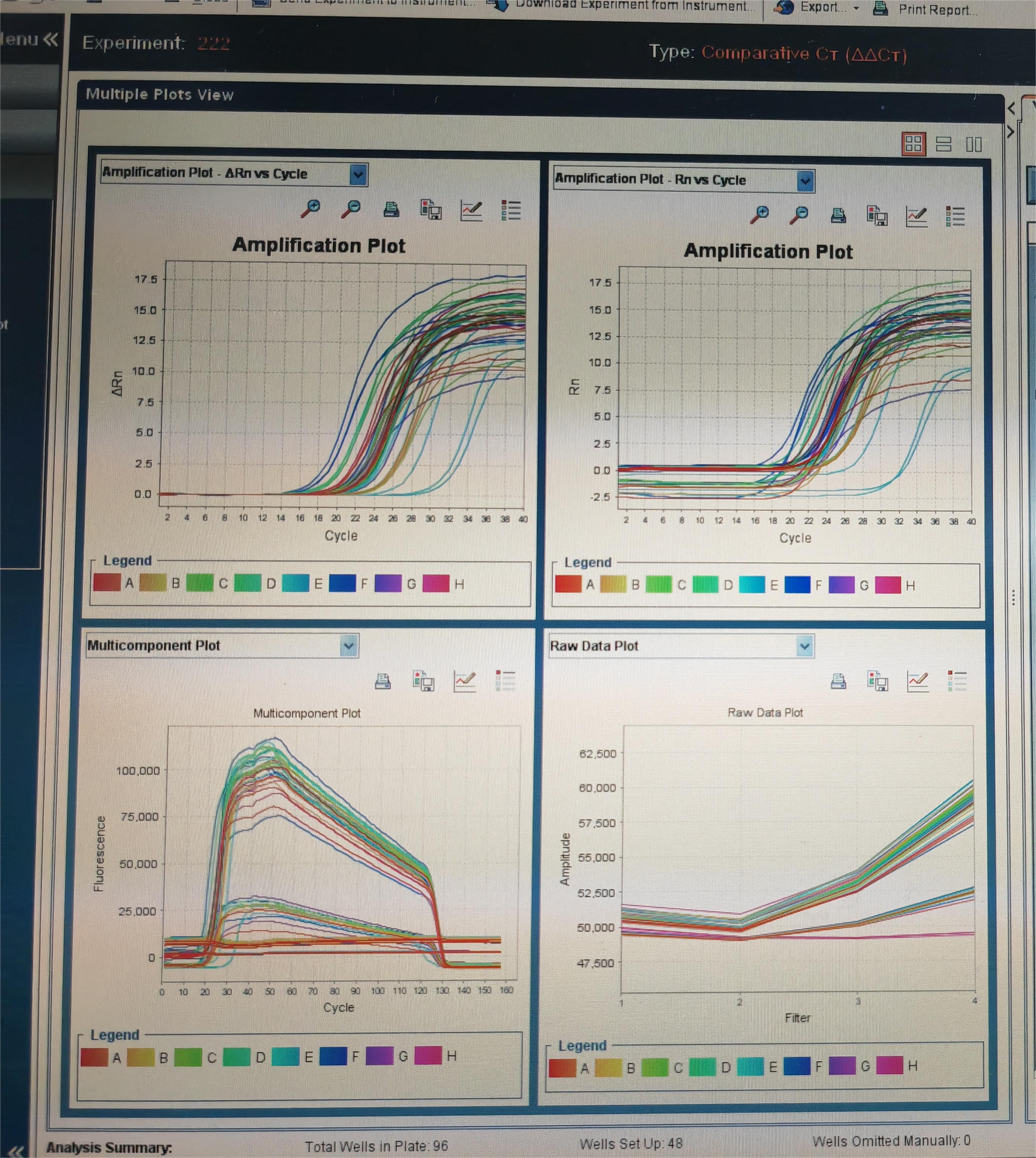

Supplement: Supplemental Information 2 [file peerj-12-18406-s002.zip › qPCR raw date/organization GAPDH FOXD1.jpg]

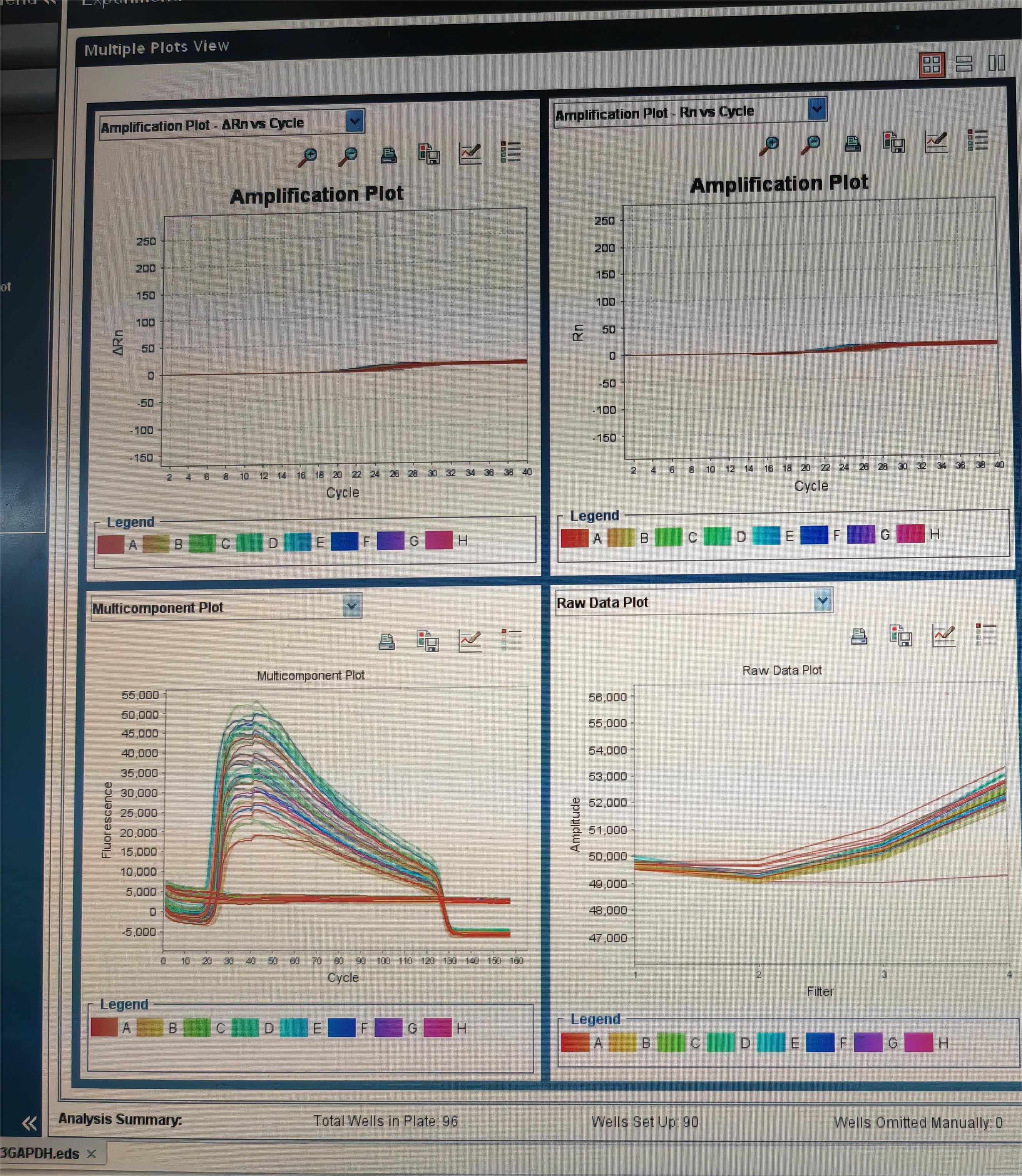

Supplement: Supplemental Information 2 [file peerj-12-18406-s002.zip › qPCR raw date/organization GAPDH FOXD4.jpg]
