# Supplementary material for: Forkhead box D subfamily genes in colorectal cancer: potential biomarkers and therapeutic targets
Source: PeerJ. 2024 Oct 29;12:e18406. doi: 10.7717/peerj.18406 (PMC11529599; doi:10.7717/peerj.18406)
Supplement: Supplemental Information 4 [file peerj-12-18406-s004.doc]

**Table S1 Baseline data of patients**

| Characteristic | FOXD1 | | | FOXD2 | | | FOXD3 | | | FOXD4 | | |
| --- | --- | --- | --- | --- | --- | --- | --- | --- | --- | --- | --- | --- |
|  | Low expression | High expression | p | Low expression | High expression | p | Low expression | High expression | p | Low expression | High expression | p |
| n | 322 | 322 |  | 322 | 322 |  | 322 | 322 |  | 322 | 322 |  |
| T stage, n (%) |  |  | 0.071 |  |  | 0.172 |  |  | 0.577 |  |  | 0.471 |
| T1 | 14 (2.2%) | 6 (0.9%) |  | 8 (1.2%) | 12 (1.9%) |  | 12 (1.9%) | 8 (1.2%) |  | 12 (1.9%) | 8 (1.2%) |  |
| T2 | 61 (9.5%) | 50 (7.8%) |  | 51 (8%) | 60 (9.4%) |  | 60 (9.4%) | 51 (8%) |  | 61 (9.5%) | 50 (7.8%) |  |
| T3 | 215 (33.5%) | 221 (34.5%) |  | 217 (33.9%) | 219 (34.2%) |  | 211 (32.9%) | 225 (35.1%) |  | 214 (33.4%) | 222 (34.6%) |  |
| T4 | 30 (4.7%) | 44 (6.9%) |  | 45 (7%) | 29 (4.5%) |  | 37 (5.8%) | 37 (5.8%) |  | 34 (5.3%) | 40 (6.2%) |  |
| N stage, n (%) |  |  | 0.475 |  |  | 0.117 |  |  | 0.003 |  |  | 0.013 |
| N0 | 180 (28.1%) | 188 (29.4%) |  | 186 (29.1%) | 182 (28.4%) |  | 204 (31.9%) | 164 (25.6%) |  | 166 (25.9%) | 202 (31.6%) |  |
| N1 | 83 (13%) | 70 (10.9%) |  | 67 (10.5%) | 86 (13.4%) |  | 70 (10.9%) | 83 (13%) |  | 89 (13.9%) | 64 (10%) |  |
| N2 | 57 (8.9%) | 62 (9.7%) |  | 67 (10.5%) | 52 (8.1%) |  | 46 (7.2%) | 73 (11.4%) |  | 65 (10.2%) | 54 (8.4%) |  |
| M stage, n (%) |  |  | 0.343 |  |  | 0.337 |  |  | 0.388 |  |  | 0.229 |
| M0 | 227 (40.2%) | 248 (44%) |  | 243 (43.1%) | 232 (41.1%) |  | 235 (41.7%) | 240 (42.6%) |  | 236 (41.8%) | 239 (42.4%) |  |
| M1 | 48 (8.5%) | 41 (7.3%) |  | 40 (7.1%) | 49 (8.7%) |  | 39 (6.9%) | 50 (8.9%) |  | 51 (9%) | 38 (6.7%) |  |
| Pathologic stage, n (%) |  |  | 0.086 |  |  | 0.704 |  |  | 0.051 |  |  | 0.004 |
| Stage I | 64 (10.3%) | 47 (7.5%) |  | 54 (8.7%) | 57 (9.1%) |  | 61 (9.8%) | 50 (8%) |  | 59 (9.5%) | 52 (8.3%) |  |
| Stage II | 105 (16.9%) | 133 (21.3%) |  | 125 (20.1%) | 113 (18.1%) |  | 131 (21%) | 107 (17.2%) |  | 98 (15.7%) | 140 (22.5%) |  |
| Stage III | 92 (14.8%) | 92 (14.8%) |  | 93 (14.9%) | 91 (14.6%) |  | 80 (12.8%) | 104 (16.7%) |  | 105 (16.9%) | 79 (12.7%) |  |
| Stage IV | 49 (7.9%) | 41 (6.6%) |  | 41 (6.6%) | 49 (7.9%) |  | 40 (6.4%) | 50 (8%) |  | 51 (8.2%) | 39 (6.3%) |  |
| Gender, n (%) |  |  | 0.155 |  |  | 0.269 |  |  | 0.343 |  |  | 0.343 |
| Female | 141 (21.9%) | 160 (24.8%) |  | 143 (22.2%) | 158 (24.5%) |  | 157 (24.4%) | 144 (22.4%) |  | 144 (22.4%) | 157 (24.4%) |  |
| Male | 181 (28.1%) | 162 (25.2%) |  | 179 (27.8%) | 164 (25.5%) |  | 165 (25.6%) | 178 (27.6%) |  | 178 (27.6%) | 165 (25.6%) |  |
| Age, n (%) |  |  | 0.014 |  |  | 0.301 |  |  | 0.937 |  |  | 0.691 |
| <=65 | 154 (23.9%) | 122 (18.9%) |  | 131 (20.3%) | 145 (22.5%) |  | 137 (21.3%) | 139 (21.6%) |  | 135 (21%) | 141 (21.9%) |  |
| >65 | 168 (26.1%) | 200 (31.1%) |  | 191 (29.7%) | 177 (27.5%) |  | 185 (28.7%) | 183 (28.4%) |  | 187 (29%) | 181 (28.1%) |  |
| Lymphatic invasion, n (%) |  |  | 0.577 |  |  | 0.722 |  |  | < 0.001 |  |  | 0.318 |
| No | 180 (30.9%) | 170 (29.2%) |  | 177 (30.4%) | 173 (29.7%) |  | 192 (33%) | 158 (27.1%) |  | 168 (28.9%) | 182 (31.3%) |  |
| Yes | 113 (19.4%) | 119 (20.4%) |  | 113 (19.4%) | 119 (20.4%) |  | 94 (16.2%) | 138 (23.7%) |  | 122 (21%) | 110 (18.9%) |  |
| History of colon polyps, n (%) |  |  | 0.040 |  |  | 0.080 |  |  | 0.249 |  |  | 0.202 |
| No | 204 (36.8%) | 173 (31.2%) |  | 176 (31.7%) | 201 (36.2%) |  | 195 (35.1%) | 182 (32.8%) |  | 182 (32.8%) | 195 (35.1%) |  |
| Yes | 79 (14.2%) | 99 (17.8%) |  | 98 (17.7%) | 80 (14.4%) |  | 82 (14.8%) | 96 (17.3%) |  | 97 (17.5%) | 81 (14.6%) |  |
| OS event, n (%) |  |  | 0.049 |  |  | 0.115 |  |  | 0.237 |  |  | 0.049 |
| Alive | 268 (41.6%) | 247 (38.4%) |  | 249 (38.7%) | 266 (41.3%) |  | 264 (41%) | 251 (39%) |  | 268 (41.6%) | 247 (38.4%) |  |
| Dead | 54 (8.4%) | 75 (11.6%) |  | 73 (11.3%) | 56 (8.7%) |  | 58 (9%) | 71 (11%) |  | 54 (8.4%) | 75 (11.6%) |  |
| Age, meidan (IQR) | 66 (57, 75) | 69 (60, 77) | 0.014 | 69 (59, 77) | 67 (57, 74) | 0.046 | 68 (58, 75) | 68 (58, 77) | 0.803 | 68 (58, 75) | 68 (58, 77) | 0.452 |
