# Supplementary material for: Forkhead box D subfamily genes in colorectal cancer: potential biomarkers and therapeutic targets
Source: PeerJ. 2024 Oct 29;12:e18406. doi: 10.7717/peerj.18406 (PMC11529599; doi:10.7717/peerj.18406)
Supplement: Supplemental Information 5 [file peerj-12-18406-s005.doc]

**Table S2 Univariate and multivariate Cox regression analysis of FOXD subfamily**

**FOXD1**

| Characteristics | Total(N) | Univariate analysis | |  | Multivariate analysis | |
| --- | --- | --- | --- | --- | --- | --- |
|  |  | Hazard ratio (95% CI) | P value |  | Hazard ratio (95% CI) | P value |
| T stage (T3&T4 vs. T1&T2) | 640 | 2.468 (1.327-4.589) | **0.004** |  | 2.245 (0.949-5.312) | 0.066 |
| N stage (N1&N2 vs. N0) | 639 | 2.627 (1.831-3.769) | **<0.001** |  | 0.323 (0.108-0.968) | **0.044** |
| M stage (M1 vs. M0) | 563 | 3.989 (2.684-5.929) | **<0.001** |  | 2.509 (1.484-4.239) | **<0.001** |
| Pathologic stage (Stage III&Stage IV vs. Stage I&Stage II) | 622 | 2.988 (2.042-4.372) | **<0.001** |  | 6.081 (1.831-20.195) | **0.003** |
| Gender (Male vs. Female) | 643 | 1.054 (0.744-1.491) | 0.769 |  |  |  |
| Age (>65 vs. <=65) | 643 | 1.939 (1.320-2.849) | **<0.001** |  | 2.777 (1.714-4.498) | **<0.001** |
| History of colon polyps (Yes vs. No) | 554 | 0.789 (0.496-1.257) | 0.319 |  |  |  |
| Lymphatic invasion (Yes vs. No) | 581 | 2.144 (1.476-3.114) | **<0.001** |  | 1.493 (0.957-2.329) | 0.077 |
| FOXD1 (High vs. Low) | 643 | 1.502 (1.058-2.132) | **0.023** |  | 1.563 (1.018-2.399) | **0.041** |

**FOXD2**

| Characteristics | Total(N) | Univariate analysis | |  | Multivariate analysis | |
| --- | --- | --- | --- | --- | --- | --- |
|  |  | Hazard ratio (95% CI) | P value |  | Hazard ratio (95% CI) | P value |
| T stage (T3&T4 vs. T1&T2) | 640 | 2.468 (1.327-4.589) | **0.004** |  | 2.384 (1.002-5.674) | 0.050 |
| N stage (N1&N2 vs. N0) | 639 | 2.627 (1.831-3.769) | **<0.001** |  | 0.324 (0.108-0.971) | **0.044** |
| M stage (M1 vs. M0) | 563 | 3.989 (2.684-5.929) | **<0.001** |  | 2.251 (1.345-3.767) | **0.002** |
| Pathologic stage (Stage III&Stage IV vs. Stage I&Stage II) | 622 | 2.988 (2.042-4.372) | **<0.001** |  | 6.235 (1.875-20.741) | **0.003** |
| Gender (Male vs. Female) | 643 | 1.054 (0.744-1.491) | 0.769 |  |  |  |
| Age (>65 vs. <=65) | 643 | 1.939 (1.320-2.849) | **<0.001** |  | 2.903 (1.800-4.682) | **<0.001** |
| History of colon polyps (Yes vs. No) | 554 | 0.789 (0.496-1.257) | 0.319 |  |  |  |
| Lymphatic invasion (Yes vs. No) | 581 | 2.144 (1.476-3.114) | **<0.001** |  | 1.525 (0.974-2.388) | 0.065 |
| FOXD2 (High vs. Low) | 643 | 0.813 (0.574-1.153) | 0.246 |  |  |  |

**FOXD3**

| Characteristics | Total(N) | Univariate analysis | |  | Multivariate analysis | |
| --- | --- | --- | --- | --- | --- | --- |
|  |  | Hazard ratio (95% CI) | P value |  | Hazard ratio (95% CI) | P value |
| T stage (T3&T4 vs. T1&T2) | 640 | 2.468 (1.327-4.589) | **0.004** |  | 2.494 (1.038-5.992) | **0.041** |
| N stage (N1&N2 vs. N0) | 639 | 2.627 (1.831-3.769) | **<0.001** |  | 0.301 (0.099-0.916) | **0.034** |
| M stage (M1 vs. M0) | 563 | 3.989 (2.684-5.929) | **<0.001** |  | 2.294 (1.370-3.842) | **0.002** |
| Pathologic stage (Stage III&Stage IV vs. Stage I&Stage II) | 622 | 2.988 (2.042-4.372) | **<0.001** |  | 6.388 (1.912-21.344) | **0.003** |
| Gender (Male vs. Female) | 643 | 1.054 (0.744-1.491) | 0.769 |  |  |  |
| Age (>65 vs. <=65) | 643 | 1.939 (1.320-2.849) | **<0.001** |  | 2.946 (1.826-4.753) | **<0.001** |
| History of colon polyps (Yes vs. No) | 554 | 0.789 (0.496-1.257) | 0.319 |  |  |  |
| Lymphatic invasion (Yes vs. No) | 581 | 2.144 (1.476-3.114) | **<0.001** |  | 1.454 (0.923-2.290) | 0.106 |
| FOXD3 (High vs. Low) | 643 | 1.460 (1.028-2.074) | **0.035** |  | 1.331 (0.865-2.048) | 0.194 |

**FOXD4**

| Characteristics | Total(N) | Univariate analysis | |  | Multivariate analysis | |
| --- | --- | --- | --- | --- | --- | --- |
|  |  | Hazard ratio (95% CI) | P value |  | Hazard ratio (95% CI) | P value |
| T stage (T3&T4 vs. T1&T2) | 640 | 2.468 (1.327-4.589) | **0.004** |  | 2.207 (0.931-5.228) | 0.072 |
| N stage (N1&N2 vs. N0) | 639 | 2.627 (1.831-3.769) | **<0.001** |  | 0.352 (0.118-1.052) | 0.061 |
| M stage (M1 vs. M0) | 563 | 3.989 (2.684-5.929) | **<0.001** |  | 2.358 (1.407-3.951) | **0.001** |
| Pathologic stage (Stage III&Stage IV vs. Stage I&Stage II) | 622 | 2.988 (2.042-4.372) | **<0.001** |  | 6.106 (1.845-20.215) | **0.003** |
| Gender (Male vs. Female) | 643 | 1.054 (0.744-1.491) | 0.769 |  |  |  |
| Age (>65 vs. <=65) | 643 | 1.939 (1.320-2.849) | **<0.001** |  | 2.948 (1.826-4.759) | **<0.001** |
| History of colon polyps (Yes vs. No) | 554 | 0.789 (0.496-1.257) | 0.319 |  |  |  |
| Lymphatic invasion (Yes vs. No) | 581 | 2.144 (1.476-3.114) | **<0.001** |  | 1.492 (0.953-2.337) | 0.081 |
| FOXD4 (High vs. Low) | 643 | 1.431 (1.007-2.032) | **0.045** |  | 1.535 (1.008-2.336) | **0.046** |
