# Supplementary material for: Forkhead box D subfamily genes in colorectal cancer: potential biomarkers and therapeutic targets
Source: PeerJ. 2024 Oct 29;12:e18406. doi: 10.7717/peerj.18406 (PMC11529599; doi:10.7717/peerj.18406)
Supplement: Supplemental Information 6 [file peerj-12-18406-s006.doc]

**Table S3 The results of GO enrichment analysis**

| **Gene** | **ONTOLOGY** | **ID** | **Description** | **pvalue** | **p.adjust** | **qvalue** | **Count** |
| --- | --- | --- | --- | --- | --- | --- | --- |
| FOXD1 | BP | GO:0001656 | metanephros development | 8.37E-11 | 3.56E-07 | 3.01E-07 | 15 |
| FOXD1 | BP | GO:0003002 | regionalization | 1.70E-09 | 3.62E-06 | 3.06E-06 | 26 |
| FOXD1 | BP | GO:0070268 | cornification | 1.66E-08 | 1.96E-05 | 1.66E-05 | 14 |
| FOXD1 | BP | GO:0007389 | pattern specification process | 1.85E-08 | 1.96E-05 | 1.66E-05 | 28 |
| FOXD1 | BP | GO:0090183 | regulation of kidney development | 5.56E-08 | 4.73E-05 | 4.00E-05 | 10 |
| FOXD1 | CC | GO:0001533 | cornified envelope | 1.65E-05 | 0.006132 | 0.005535 | 7 |
| FOXD1 | MF | GO:0004867 | serine-type endopeptidase inhibitor activity | 9.58E-06 | 0.006427 | 0.005767 | 10 |
| FOXD1 | MF | GO:0005200 | structural constituent of cytoskeleton | 9.87E-05 | 0.033109 | 0.029709 | 9 |
| FOXD2 | BP | GO:0070268 | cornification | 5.13E-16 | 2.45E-12 | 2.15E-12 | 25 |
| FOXD2 | BP | GO:0019730 | antimicrobial humoral response | 3.69E-12 | 8.80E-09 | 7.73E-09 | 23 |
| FOXD2 | BP | GO:0030216 | keratinocyte differentiation | 5.73E-12 | 9.10E-09 | 7.99E-09 | 34 |
| FOXD2 | BP | GO:0008544 | epidermis development | 3.87E-11 | 4.61E-08 | 4.05E-08 | 42 |
| FOXD2 | BP | GO:0043588 | skin development | 5.83E-11 | 5.56E-08 | 4.88E-08 | 39 |
| FOXD2 | CC | GO:0001533 | cornified envelope | 3.42E-08 | 1.51E-05 | 1.34E-05 | 11 |
| FOXD2 | CC | GO:0097209 | epidermal lamellar body | 8.71E-05 | 0.019246 | 0.017051 | 3 |
| FOXD2 | CC | GO:0070820 | tertiary granule | 0.000232 | 0.034202 | 0.030301 | 14 |
| FOXD2 | CC | GO:0031225 | anchored component of membrane | 0.000357 | 0.039443 | 0.034944 | 14 |
| FOXD2 | MF | GO:0004252 | serine-type endopeptidase activity | 3.28E-08 | 1.21E-05 | 1.03E-05 | 20 |
| FOXD2 | MF | GO:0008236 | serine-type peptidase activity | 3.76E-08 | 1.21E-05 | 1.03E-05 | 21 |
| FOXD2 | MF | GO:0048018 | receptor ligand activity | 5.25E-08 | 1.21E-05 | 1.03E-05 | 36 |
| FOXD2 | MF | GO:0017171 | serine hydrolase activity | 5.45E-08 | 1.21E-05 | 1.03E-05 | 21 |
| FOXD2 | MF | GO:0030546 | signaling receptor activator activity | 6.78E-08 | 1.21E-05 | 1.03E-05 | 36 |
| FOXD3 | BP | GO:0006936 | muscle contraction | 1.49E-14 | 3.98E-11 | 3.12E-11 | 41 |
| FOXD3 | BP | GO:0003012 | muscle system process | 1.51E-14 | 3.98E-11 | 3.12E-11 | 47 |
| FOXD3 | BP | GO:0042391 | regulation of membrane potential | 7.27E-13 | 1.28E-09 | 1.00E-09 | 43 |
| FOXD3 | BP | GO:0007409 | axonogenesis | 4.49E-11 | 5.91E-08 | 4.63E-08 | 42 |
| FOXD3 | BP | GO:0050804 | modulation of chemical synaptic transmission | 9.51E-11 | 8.92E-08 | 6.99E-08 | 40 |
| FOXD3 | CC | GO:0062023 | collagen-containing extracellular matrix | 2.71E-16 | 1.34E-13 | 9.79E-14 | 48 |
| FOXD3 | CC | GO:0097060 | synaptic membrane | 1.41E-12 | 3.49E-10 | 2.54E-10 | 40 |
| FOXD3 | CC | GO:0099240 | intrinsic component of synaptic membrane | 7.77E-12 | 1.28E-09 | 9.35E-10 | 25 |
| FOXD3 | CC | GO:0098978 | glutamatergic synapse | 2.16E-10 | 2.68E-08 | 1.95E-08 | 35 |
| FOXD3 | CC | GO:0099699 | integral component of synaptic membrane | 4.01E-10 | 3.97E-08 | 2.90E-08 | 22 |
| FOXD3 | MF | GO:0005201 | extracellular matrix structural constituent | 1.15E-08 | 9.48E-06 | 8.01E-06 | 21 |
| FOXD3 | MF | GO:0005539 | glycosaminoglycan binding | 3.97E-08 | 1.63E-05 | 1.38E-05 | 24 |
| FOXD3 | MF | GO:0048018 | receptor ligand activity | 1.28E-07 | 3.37E-05 | 2.85E-05 | 36 |
| FOXD3 | MF | GO:0030546 | signaling receptor activator activity | 1.64E-07 | 3.37E-05 | 2.85E-05 | 36 |
| FOXD3 | MF | GO:0008201 | heparin binding | 2.85E-07 | 4.68E-05 | 3.96E-05 | 19 |
| FOXD4 | BP | GO:0000353 | formation of quadruple SL/U4/U5/U6 snRNP | 4.98E-10 | 2.80E-07 | 2.66E-07 | 6 |
| FOXD4 | BP | GO:0000365 | mRNA trans splicing, via spliceosome | 4.98E-10 | 2.80E-07 | 2.66E-07 | 6 |
| FOXD4 | BP | GO:0045291 | mRNA trans splicing, SL addition | 4.98E-10 | 2.80E-07 | 2.66E-07 | 6 |
| FOXD4 | BP | GO:0000244 | spliceosomal tri-snRNP complex assembly | 3.82E-09 | 1.61E-06 | 1.53E-06 | 7 |
| FOXD4 | BP | GO:0000387 | spliceosomal snRNP assembly | 6.18E-07 | 0.000209 | 0.000198 | 7 |
| FOXD4 | CC | GO:0097525 | spliceosomal snRNP complex | 1.57E-09 | 3.63E-07 | 3.28E-07 | 11 |
| FOXD4 | CC | GO:0030532 | small nuclear ribonucleoprotein complex | 3.03E-09 | 3.63E-07 | 3.28E-07 | 11 |
| FOXD4 | CC | GO:0120114 | Sm-like protein family complex | 9.93E-09 | 7.94E-07 | 7.17E-07 | 11 |
| FOXD4 | MF | GO:0030627 | pre-mRNA 5'-splice site binding | 7.51E-07 | 0.000225 | 0.000195 | 5 |
| FOXD4 | MF | GO:0036002 | pre-mRNA binding | 0.000142 | 0.021332 | 0.018488 | 5 |
