# Supplementary material for: Forkhead box D subfamily genes in colorectal cancer: potential biomarkers and therapeutic targets
Source: PeerJ. 2024 Oct 29;12:e18406. doi: 10.7717/peerj.18406 (PMC11529599; doi:10.7717/peerj.18406)
Supplement: Supplemental Information 7 [file peerj-12-18406-s007.doc]

**Table S4 KEGG pathway enrichment analysis**

| **Gene** | **ID** | **Description** | **pvalue** | **p.adjust** | **qvalue** | **Count** |
| --- | --- | --- | --- | --- | --- | --- |
| FOXD2 | hsa04975 | Fat digestion and absorption | 2.30E-06 | 0.000573 | 0.000523 | 9 |
| FOXD2 | hsa04061 | Viral protein interaction with cytokine and cytokine receptor | 0.000107 | 0.010626 | 0.009703 | 11 |
| FOXD2 | hsa04972 | Pancreatic secretion | 0.000128 | 0.010626 | 0.009703 | 11 |
| FOXD2 | hsa05150 | Staphylococcus aureus infection | 0.000345 | 0.021457 | 0.019593 | 10 |
| FOXD2 | hsa04060 | Cytokine-cytokine receptor interaction | 0.000625 | 0.027278 | 0.024908 | 19 |
| FOXD2 | hsa04742 | Taste transduction | 0.000657 | 0.027278 | 0.024908 | 9 |
| FOXD2 | hsa00910 | Nitrogen metabolism | 0.001086 | 0.038638 | 0.035281 | 4 |
| FOXD2 | hsa04657 | IL-17 signaling pathway | 0.001252 | 0.038974 | 0.035588 | 9 |
| FOXD2 | hsa04612 | Antigen processing and presentation | 0.001489 | 0.041196 | 0.037617 | 8 |
| FOXD2 | hsa04915 | Estrogen signaling pathway | 0.001706 | 0.04248 | 0.038789 | 11 |
| FOXD2 | hsa00512 | Mucin type O-glycan biosynthesis | 0.003114 | 0.069963 | 0.063885 | 5 |
| FOXD2 | hsa04080 | Neuroactive ligand-receptor interaction | 0.003372 | 0.069963 | 0.063885 | 19 |
| FOXD2 | hsa04650 | Natural killer cell mediated cytotoxicity | 0.003752 | 0.071869 | 0.065626 | 10 |
| FOXD2 | hsa04970 | Salivary secretion | 0.004516 | 0.080316 | 0.073339 | 8 |
| FOXD3 | hsa04020 | Calcium signaling pathway | 4.67E-08 | 7.19E-06 | 6.58E-06 | 24 |
| FOXD3 | hsa04080 | Neuroactive ligand-receptor interaction | 6.48E-08 | 7.19E-06 | 6.58E-06 | 29 |
| FOXD3 | hsa04512 | ECM-receptor interaction | 0.000156 | 0.008998 | 0.008234 | 10 |
| FOXD3 | hsa04360 | Axon guidance | 0.000162 | 0.008998 | 0.008234 | 15 |
| FOXD3 | hsa05150 | Staphylococcus aureus infection | 0.000321 | 0.014263 | 0.013053 | 10 |
| FOXD3 | hsa04911 | Insulin secretion | 0.000617 | 0.022824 | 0.020887 | 9 |
| FOXD3 | hsa04310 | Wnt signaling pathway | 0.000721 | 0.022868 | 0.020927 | 13 |
| FOXD3 | hsa04514 | Cell adhesion molecules | 0.000899 | 0.024947 | 0.022829 | 12 |
| FOXD3 | hsa04024 | cAMP signaling pathway | 0.001158 | 0.028559 | 0.026135 | 15 |
| FOXD3 | hsa04510 | Focal adhesion | 0.001434 | 0.031842 | 0.02914 | 14 |
| FOXD3 | hsa04974 | Protein digestion and absorption | 0.002238 | 0.045169 | 0.041336 | 9 |
| FOXD4 | hsa04970 | Salivary secretion | 0.000251 | 0.032401 | 0.029083 | 5 |
| FOXD4 | hsa04727 | GABAergic synapse | 0.002126 | 0.099157 | 0.089003 | 4 |
| FOXD4 | hsa05032 | Morphine addiction | 0.002306 | 0.099157 | 0.089003 | 4 |
