# Supplementary material for: Forkhead box D subfamily genes in colorectal cancer: potential biomarkers and therapeutic targets
Source: PeerJ. 2024 Oct 29;12:e18406. doi: 10.7717/peerj.18406 (PMC11529599; doi:10.7717/peerj.18406)
Supplement: Supplemental Information 8 [file peerj-12-18406-s008.doc]

**Table S5 The results of Gene Set Enrichment Analysis (GSEA).**

| **ID** | **enrichmentScore** | **NES** | **pvalue** | **p.adjust** | **qvalues** |
| --- | --- | --- | --- | --- | --- |
| KEGG_CYTOKINE_CYTOKINE_RECEPTOR_INTERACTION | 0.56878 | 1.870756 | 1.00E-10 | 1.83E-08 | 1.32E-08 |
| KEGG_NATURAL_KILLER_CELL_MEDIATED_CYTOTOXICITY | 0.6158 | 1.952165 | 5.75E-09 | 3.51E-07 | 2.52E-07 |
| KEGG_FOCAL_ADHESION | 0.646893 | 1.801249 | 1.00E-10 | 4.50E-09 | 2.74E-09 |
| KEGG_RIBOSOME | -0.63429 | -2.78166 | 1.00E-10 | 4.50E-09 | 2.74E-09 |
| KEGG_OXIDATIVE_PHOSPHORYLATION | -0.46546 | -2.04335 | 1.63E-06 | 2.67E-05 | 1.62E-05 |
| KEGG_ECM_RECEPTOR_INTERACTION | 0.515054 | 2.113675 | 6.53E-07 | 5.97E-05 | 5.02E-05 |
