# Supplementary material for: Forkhead box D subfamily genes in colorectal cancer: potential biomarkers and therapeutic targets
Source: PeerJ. 2024 Oct 29;12:e18406. doi: 10.7717/peerj.18406 (PMC11529599; doi:10.7717/peerj.18406)
Supplement: Supplemental Information 9 [file peerj-12-18406-s009.doc]

**Table S6 A** **list of primer sequences**

| Gene name |  | Primer sequences(5’-3’) |  |  |
| --- | --- | --- | --- | --- |
| FOXD1 |  | F:TATGACCCTGAGCACTGAGAT |  |  |
|  |  | R: CCTCCTCTCCTCGTCTTCTT |  |  |
| FOXD2 |  | F: CTGCGCCAAAGCCTTCTACG |  |  |
|  |  | R: GTGGCCCATGATGTGGTCTAT |  |  |
| FOXD3 |  | F: AGCAAGCCCAAGAATAGC |  |  |
|  |  | R: TCCAGGGTCCAGTAGTTG |  |  |
| FOXD4 |  | F: TCATTAGTGACCGCTTCCC |  |  |
|  |  | R: TCCAGGCTCCAGTAGTTGC |  |  |
